# Dysregulation of Gene Expression of Key Signaling Mediators in PBMCs from People with Type 2 Diabetes Mellitus

**DOI:** 10.3390/ijms24032732

**Published:** 2023-02-01

**Authors:** Nilofer Qureshi, Julia Desousa, Adeela Z. Siddiqui, Betty M. Drees, David C. Morrison, Asaf A. Qureshi

**Affiliations:** 1 Department of Biomedical Sciences, Shock/Trauma Research Center, School of Medicine, University of Missouri-Kansas City, 2411 Holmes Street, Kansas City, MO 64108, USA; 2 Department of Pharmacology/Toxicology, School of Pharmacy, University of Missouri-Kansas City, Kansas City, MO 64108, USA; 3 Internal Medicine, School of Medicine, University of Missouri-Kansas City, 2411 Holmes Street, Kansas City, MO 64108, USA

**Keywords:** type 2 diabetes, LPS, signal transduction, resveratrol, lectins, cytokines, NO, IFN-γ, NGS

## Abstract

Diabetes is currently the fifth leading cause of death by disease in the USA. The underlying mechanisms for type 2 Diabetes Mellitus (DM2) and the enhanced susceptibility of such patients to inflammatory disorders and infections remain to be fully defined. We have recently shown that peripheral blood mononuclear cells (PBMCs) from non-diabetic people upregulate expression of inflammatory genes in response to proteasome modulators, such as bacterial lipopolysaccharide (LPS) and soybean lectin (LEC); in contrast, resveratrol (RES) downregulates this response. We hypothesized that LPS and LEC will also elicit a similar upregulation of gene expression of key signaling mediators in (PBMCs) from people with type 2 diabetes (PwD2, with chronic inflammation) ex vivo. Unexpectedly, using next generation sequencing (NGS), we show for the first time, that PBMCs from PwD2 failed to elicit a robust LPS- and LEC-induced gene expression of proteasome subunit LMP7 (*PSMB8*) and mediators of T cell signaling that were observed in non-diabetic controls. These repressed genes included: *PSMB8*, *PSMB9*, interferon-γ, interferon-*λ,* signal-transducer-and-activator-of-transcription-1 (*STAT1*), human leukocyte antigen (*HLA DQB1*, *HLA DQA1*) molecules, interleukin 12A, tumor necrosis factor-α, transporter associated with antigen processing 1 (*TAP1*), and several others, which showed a markedly weak upregulation with toxins in PBMCs from PwD2, as compared to those from non-diabetics. Resveratrol (proteasome inhibitor) further downregulated the gene expression of these inflammatory mediators in PBMCs from PwD2. These results might explain why PwD2 may be susceptible to infectious disease. LPS and toxins may be leading to inflammation, insulin resistance, and thus, metabolic changes in the host cells.

## 1. Introduction

Diabetes mellitus (DM) is currently the fifth leading cause of death by disease in the U.S. As of 2015, ~415 million individuals worldwide (8.3% of adults) have been diagnosed with diabetes. Many diabetes investigators have focused on reducing insulin resistance and reducing elevated circulating glucose levels to impact the complications, morbidity, and mortality of people with type 2 diabetes mellitus (PwD2), which generally develop later in life and are associated with diet and lifestyle. The role of PwD2 in increased susceptibility and worse outcomes of infectious diseases has been well documented [1,2,3,4,5,6,7,8,9]. It has also been implicated in the higher risks and mortality of viral diseases, such as COVID-19 [1,2], AIDS, SARS, Herpes Simplex, and enterovirus. The first line of treatment for PwD2 is metformin, as suggested by the American Diabetes Association ADA [10,11]. However, the mechanisms for the susceptibility of PwD2 to inflammatory and infectious diseases are not well-understood.

PwD2 are at increased risk for lower respiratory tract, urinary tract, skin, and mucous membrane infections [1,2,3,4,5,6,7,8,9]. Generally, infectious diseases are more frequent and/or serious in PwD2, affecting all organs and systems [1,2,3,4,5,6,7,8,9]. Koh et al. have reviewed and discussed the immune response in diabetes and its potential contribution to the pathogenesis of sepsis and insulin resistance, which are common features of PwD2 [9]. The human gut has a wide variety of microbiota and Saad et al. have reviewed the link between the intestinal flora, inflammation via Toll receptors, and insulin resistance [12]. However, new approaches are needed to study immune function in diabetes and dietary components so that therapeutic strategies to improve immune function, reduce the risk of infections, and improve outcomes can be designed.

PwD2 have been reported to manifest primarily an M1 macrophage inflammatory cytokine response, as reported by several investigators [13,14,15,16,17,18,19]. There is ample evidence for a prominent role for host macrophages/monocytes (Mφ/MO) in almost all aspects of the host immune response. There are at least two distinct macrophage phenotypes, distinguished by differentiation state, referred to as M1 (high inflammation), and M2 (low inflammation) [20]. Following activation of M1 phenotype, cells may further differentiate to an M2 phenotype in response to newly generated cytokines (e.g., IL-4, IL-13) and environmental factors which are designed to promote cell proliferation and tissue repair. M1 and M2 macrophages function to promote the differentiation of Th1/Th2 (helper T cells), such that Th1 induces IFN-γ (M1 macrophages are useful for microbicidal functions), while Th2 cells induce IL-4, IL-5 and IL-13 (M2 macrophages are useful for recovery and repair) [21,22,23,24,25]. The underlying mechanisms of the action of toxins in PBMCs (containing monocytes, dendritic cells, and T cells) from PwD2 have not been investigated in detail in PwD2.

Our group and others have been actively addressing the role of the inflammatory responses induced by lipopolysaccharides (LPS, activator of inflammation) and other microbial products as prototypes in mouse macrophages and human monocytes. One of the mechanisms for LPS action involves the ubiquitin proteasome system (UPS), which is responsible for the degradation of cellular proteins, transcription factors and controls several functions of the cell. This is accomplished by at least six proteasome protease subunits [26,27,28,29,30,31,32,33,34,35,36]. The IFN-γ or LPS can lead to the downregulation of the X (chymotrypsin-like), Y (post acidic), Z subunits (trypsin-like) (constitutive subunits, basal state) and upregulation of LMP7 (chymotrypsin-like), LMP2 (post acidic), and LMP10 (trypsin-like) (low molecular wt. proteins, inducible immunosubunits) in the cells [26,35,36,37]. All cells contain proteasomes, but the hematopoietic cells contain predominantly LMP7 and LMP2 immunosubunits, compared to the non-hematopoietic cells. Thus, proteasomes can control LPS-induced multiple-signaling pathways in a cell by changing its subunits and proteolytic activities at precise times during the early degradation of transcription factors, cytokine induction, and inflammatory processes [29,30]. Initially, LPS causes a cytokine storm via activation of Toll receptors and induction of proinflammatory cytokines due to the activation of transcription factors, such as NF-κB, via cleavage of IκB by the proteasome. This is followed by the increased gene expression of enzymes involved in metabolism, cell cycle and immune function. Since the proteasome activities are involved in immune function, then one should be able to change the gene expression of mediators and proteins in signaling pathways using proteasome activators/inhibitors with differing degradative properties [29,30]. Proteins are also degraded by the endosomal/lysosomal system.

LPS (from bacteria) activate most of the inflammatory activities in non-diabetic controls. Resveratrol (RES, a flavonoid) [38,39,40,41,42,43,44,45,46,47,48] functions as a proteasome inhibitor and inhibits chymotrypsin-like protease activity/expression of predominantly the LMP7 (*PSMB8*) and LMP2 (*PSMB9*) subunits in human monocytes (degrade at tryptophan, tyrosine, and post-acidic residues such as aspartate and glutamate) [38,39,40]. Soybean lectin is a plant toxin (LEC) and we have also recently identified this as a prototype activator of inflammation because it can induce gene expression of macrophage and T-cell cytokines *IFN-γ*, *IL-4*, *IL-2*, and LMP7 (*PSMB8*) in non-diabetic controls just like LPS. The mechanisms by which plant products, such as RES (anti-inflammatory compound) and LEC (inflammatory compound), affect the gene expression of mediators in signaling pathways in human PBMCs of PwD2 are presently not understood [41,42,43,44,45,46,47,48,49,50,51,52,53,54].

We have recently reported that RES and LEC differentially affect LPS-induced pathways in non-diabetic controls via repression or induction of *PSMB8*, respectively [54]. Our present study was based on the hypothesis that PBMCs from non-diabetics and PwD2 would be differentially responsive to inducing the gene expression of mediators involved in signaling pathways with LPS (bacterial) and plant soybean lectins (lectins are also present in plants, bacteria, and viruses) ex vivo, being metabolically different. This would lead to an alternative modulation in gene expression of insulin receptors, glucose transporters, proteasome subunits, and mediators involved in the signaling pathways (and perhaps leading to insulin-resistance) and causing disease. Therefore, to test this hypothesis, we determined the extent to which proteasome modulators, RES and LEC, play a role in regulating the gene expression of mediators of signaling pathways in PBMCs from non-diabetics and PwD2, in the presence and absence of LPS.

## 2. Results

### 2.1. Next Generation Sequencing (NGS) Profiling Experiment

PBMCs from non-diabetic controls [54] and PwD2 (age, weight, and sex-matched) were treated with or without LPS, RES, LEC10, and LEC50. PBMCs were treated with agonists to investigate if cells from PwD2 would be able to respond to LEC, LPS and RES similarly to the non-diabetic control [54]. RNA was extracted and subjected to NGS, and log2 fold ratios were uploaded onto the Ingenuity Pathway Analysis Program (Qiagen). The two objectives of this study were as follows: 1. To define gene expression differences in PBMCs between non-diabetic and PwD2 (D). The control values mentioned in our previous manuscript were used [54]; 2. To define differences in gene expression in PBMCs from PwD2 treated with proteasome modulators, LPS (DLPS), LEC (DLEC), LPS + RES (DLPS + RES), and RES (DRES) alone using NGS. Table 1 shows the number of differentially expressed genes (DEG) in RNA samples. All the genes induced/repressed are summarized in Table 1, all other tables are presented in the Supplementary Materials (SM). LPS induced 555 genes in PBMCs from non-diabetic controls, but only 377 in PwD2. Total genes modulated by RES (80 µM), LPS (10 ng/mL), and LPS + RES (10 ng/mL and 80 µM), LEC10 (10 µg/mL). LEC10 + LPS, LEC50 (50 µg/mL) and LEC50 + LPS in PBMCs of non-diabetic controls and PwD2 are listed in Table 1.

### 2.2. Expression of Functional Genes after Treatment of PBMCs from Non-Diabetic Controls and PwD2 with LPS, RES, and LEC

**Figure 1:**
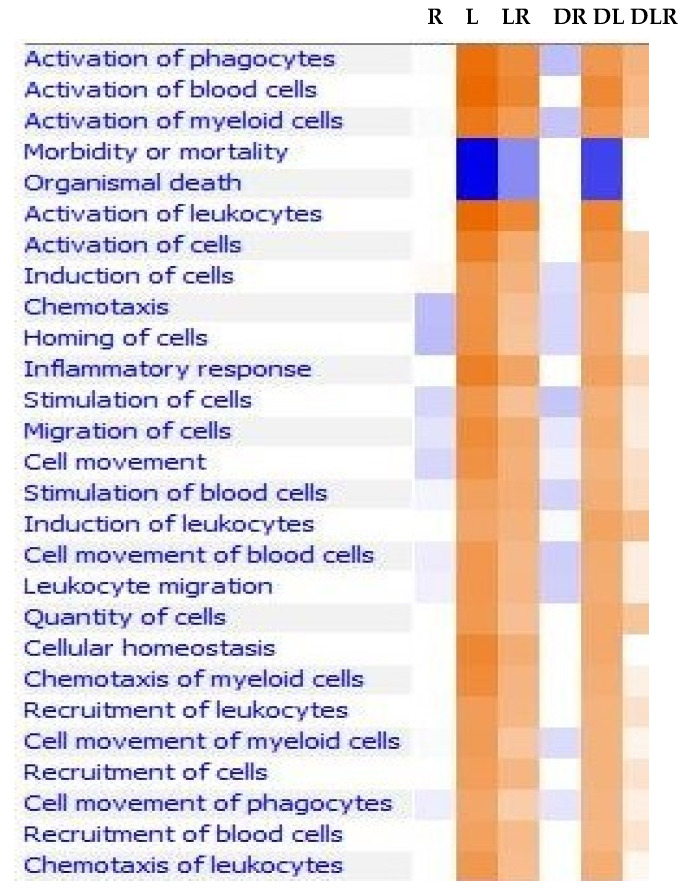
Cell functions modulated by RES, LPS, and LPS + RES in PBMCs from controls and PwD2. Human PBMCs from non-diabetic and PwD2 were treated with three different treatments and the vehicle control for 3 h. The RNA samples from controls and PwD2 were extracted from the cells and analyzed using RNAseq. These data were first extracted using the DEG analysis, followed by ingenuity pathways analysis. Cell functions for RES (R), LPS (L), LPS + RES (L + R), DRES (DR), DLPS (DL), and DLPS + RES (DL + R). Comparisons were made using their respective controls. Dark orange color denotes maximal effect, while dark blue color denotes minimal effect.

It is well-established that PwD2 have increased levels of cytokines, including TNF-α, IL-6, and IL-18 [13,14,15,16,17]. We investigated the cell functions that were upregulated by LPS and downregulated by RES in PBMCs from non-diabetics RES (R), LPS (L), and LPS-RES (LR), PwD2 RES (DR), PwD2 LPS (DL), and PwD2-LPS-RES (DLR). Respective controls for non-diabetic controls and PwD2 were used. Importantly, functional analysis of genes as related to the activation of phagocytes, blood cells, leukocytes, induction of cells, chemotaxis, homing of cells, inflammatory response, migration, cell movement, and all others (Figure 1) were robustly downregulated in RNA obtained from PBMCs of PwD2, as compared to the non-diabetic controls after treatment with LPS. RES further downregulated these genes, while LEC and LPS upregulated gene expression of genes, as described below.

**Minor differences were noted in differentially expressed gene analysis of RNA from PBMCs from non-diabetic vs. PwD2 without any LPS treatment**. The changes in gene expression values are listed in Table S1 (Supplementary Material). The downregulated genes in PwD2 samples are listed in Table S1A. Some of these genes included major histocompatibility genes *MHCII*, *HLA-DQB1*, and *HLA-DQA1* (involved in antigen presentation), histone cluster 1 (*HIST1H1C*), formyl peptide receptor 1 (*FPR1*), C-type lectin (*CLEC10A*), ATPase class 1 type 8B member 4 (*ATP8B4*), lysozyme (*LYZ*), keratin 5 (*KRT5*), phospholipase D (*PLD4*), T-cell receptor delta variable 2 (*TRDV5*), methythioribose-1-phosphate isomerase (*MRI1*), matrix metallopeptidase 7 (*MMP7*), heat shock 70 kDa protein 7 (*HSPA7*), ATPase class 1, type 88 member 4 (*ATP884*), and *TLR6*. The upregulated genes are listed in Table S1B. Some of these include Dead H box helicase 11 (*DDX11*), Claudin 5 (*CLDN5*), chemokine CXC motif (*CXCL1* ligand 1, melanoma growth), glutathione S-transferase mu 1 (*GSTM1*), major histocompatibility complex class I K (*HLA-K*), and thrombospondin 1 (*THBS1*).

**Major failure in eliciting the upregulation of gene expression of key mediators in IFN-γ signaling and other pathways in PBMCs from PwD2 compared to non-diabetic controls was observed only after treatment with LPS (*ex vivo*).** The LPS-modulated genes in PBMCs of non-diabetics and PwD2 were compared, and these are listed in Table S2A,B. Unexpectedly, there was a failure to elicit a robust upregulation of gene expression in PBMCs from PwD2 with LPS, and these genes included *IFN-γ* and its induced genes, (*IFITM3*), HLA molecules II *HLA-DQB1*, and *HLA DQA1*, chemokine genes, such as *CCL-8*, *CXCL9*, *CXCL10*, *CXCL11*, etc., as compared to non-diabetic controls. Transcription factor genes T-box 3 (*TBX-3*), gene expression for receptor for sialic acid binding Ig like lectin-like 1 sialo adhesin (*SIGLEC1*), formyl peptide receptor 1 (*FPR1*), histone cluster 1 H (*HIST1H1C* and *HIST1H1E*) were also downregulated, as compared to non-diabetic controls (Table S2A). In contrast, upregulated genes in RNA from PwD2 included chemokine ligands *CCL3L3*, *CCL4L1*, aldo-keto reductase family 1 member C2 (*AKR1C2*), DEAD/H, BOX helicase 11-like10, (*DDX11L10*), glutathione S-transferase mu 1 (*GSTM1*), mannosidase endo-alpha-like (*MANEAL*), *HLA-K*, interleukin-5 RA (*IL-5RA*), formin 1 (*FMN1*), cluster of differentiation (*CD9*), and neuropilin-1 (*NRP1*) (Table S2B).

**Figure 2:**
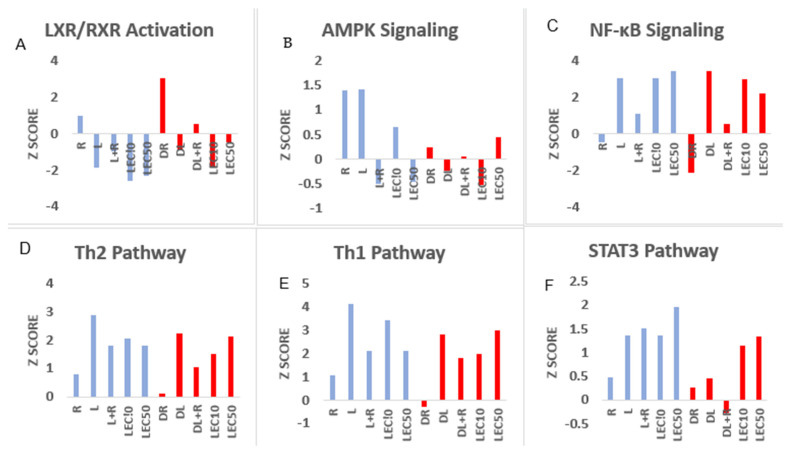
The role of RES, LPS, LPS + RES, LEC10, LEC50, on gene expression of mediators involved in signaling pathways in PBMCs from non-diabetic controls and PwD2. Human PBMCs from controls and PwD2 were treated and compared with the vehicle control for 3 h. RNA was extracted from the cells and analyzed using RNAseq. These data were first extracted using the DEG analysis, followed by ingenuity pathways analysis. Z scores are plotted against the signaling pathways. Nondiabetics RES (R); LPS (L); LPS + RES (L + R); LEC10; LEC50 (blue colored bars; Diabetics, RES (DR); LPS (DL); LPS + RES (DL + R); DLEC10 (LEC10); and DLEC50 (LEC50). (**A**–**F**) Signaling pathways modulated by LPS, LEC, RES in PBMCs from non-diabetic control (blue bars) vs. PwD2 (red colored bars).

Normally, PwD2 have been reported to have excessive inflammation in their cells followed by insulin-resistance, which leads to heart disease, cancer, and wound healing. Importantly, failure of LPS or LEC to elicit a robust upregulation of expression of genes that belonged to mediators of signaling pathways, such as pattern recognition, cardiovascular signaling, *AMPK*, *Th17* activation, *Th1* (*IFN-γ*), *Th2* (*IL-4*), *STAT3*, amyotrophic lateral sclerosis and death receptor signaling, was observed in PBMCs of PwD2, as compared to non-diabetic controls (some shown in Figure 2A–F). LPS also showed down-regulation of gene expression of mediators responsible for acute phase signaling, antioxidant action of vitamin C, and endocannabinoid cancer inhibition pathways. In contrast, gene expression of mediators involved in opioid signaling was upregulated by LPS in PBMCs of PwD2, as compared to controls.

**Further downregulation of gene expression in mediators of inflammatory signaling pathways in PBMCs from PwD2 was observed by RES treatment (alone).** To analyze RES-modulated DEG analysis, we have included inflammatory marker genes that were downregulated as shown in Table S3A. Several of the significant inflammation-linked genes were downregulated with RES alone, these include chemokine genes (*CCL2* or *MCP1*), thrombospondin 1 (*THB1*), and thrombomodulin (*THBD*); cytochrome P450 family (*CYP1B1*), early growth response 2 (*EGR2*), chemokine (*CCL7*), cannabinoid receptor 2, (*CNR2*), formyl peptide receptor 2 (*FPR2*), IL-10, aryl-hydrocarbon receptor repressor (*AHRR*), and G-protein coupled receptor 68 (*GPR68*). The expression of both inflammatory and anti-inflammatory mediator genes was downregulated when RES alone was used (a proteasome CT-like inhibitor of subunit LMP7). However, some genes were also upregulated by RES, as compared to PBMCs from PwD2, as shown in Table S3B.

**RES downregulated expression of LPS-induced genes in PBMCs from PwD2.** Twenty five of the most significant downregulated genes with LPS + RES include chemokine genes (*CCL2* or *MCP1*; *CCL7* and *CCL8*) thrombospondin 1 (*THB1*), and thrombomodulin (*THBD*); cytochrome P450 family (*CYP1B1* and *CYP1B1-AS1*), *IL-19*, gamma-glutamyltransferase-5 (*GGT5*), early growth response 2 (*EGR2*), 2′-5′-oligoadenylate synthetase 3 (*OAS3*), laminin beta 3 (*LAMB3*), IL-36 γ (*IL-36γ*), myxovirus resistance 1-interferon inducible (*MX1*), ubiquitin-specific peptidase 18, (*USP18*), and sialidase 4 (*NEU4*) (Table S4A). A major downregulation in gene expression was observed in interferon-α 5 (*IFNA5*) with RES alone. In contrast, LPS + RES also upregulated genes (Table S4A). We also observed that RES not only downregulates gene expression important to interferon-inducible genes (Tables S3A and S4A), but also other genes involved in the differentiation of cells. We have previously shown that RES acts as an inhibitor and blocks the chymotrypsin-like activity (LMP7 and to a lesser extent X) of the proteasome and downregulates the gene expression of LMP7.

**Figure 3:**
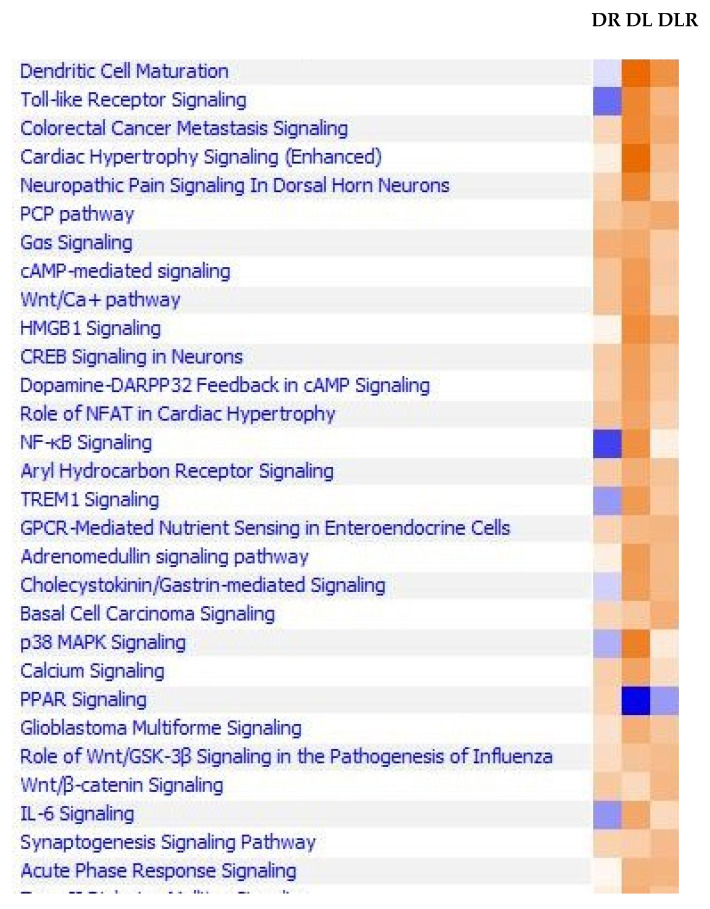
The mediators involved in signaling pathways that were downregulated by RES. The role of RES (DR), LPS (DL), and LPS + RES (DLR) on gene expression of mediators involved in the agonist-induced signal transduction in treated PBMC from PwD2 are described in the legend to Figure 2. RNAseq data were first extracted using the DEG analysis and analyzed by ingenuity pathways analysis. Z scores were plotted against the signaling pathways, where dark orange denotes maximum activation and dark blue maximum repression.

**LPS upregulated gene expression of mediators for most disease functions, whereas RES inhibited this, as shown in Figure 1.** We observed LPS-induced changes in gene expression of human leukocyte antigens (antigen presentation, HLA molecules). Although gene expression of several mediators in canonical pathways was inhibited by RES, but we have focused on IFN-γ, Th1, Th2, and pattern-recognition pathways in this manuscript (Figure 2A–F) because those were affected the most. In contrast, gene expression for key mediators in pathways for the synthesis of prostaglandins and fatty acid synthesis was upregulated by RES in human PBMC. To investigate the signaling pathways affected by RES (DR), LPS (DL), and LPS + RES (DLR), we found that LPS upregulated genes of key mediators of several inflammatory pathways that were robustly downregulated by treatment with RES in PBMCs of PwD2. These signaling pathways also included Th17 activation, HMG-B1, P38 MAPK, Th1 (IFN-γ), and Th2 (IL-4), triggering the receptor expressed on myeloid cells 1 (TREM1), IL-6, and neuroinflammation, as shown in Figure 3.

**Figure 4:**
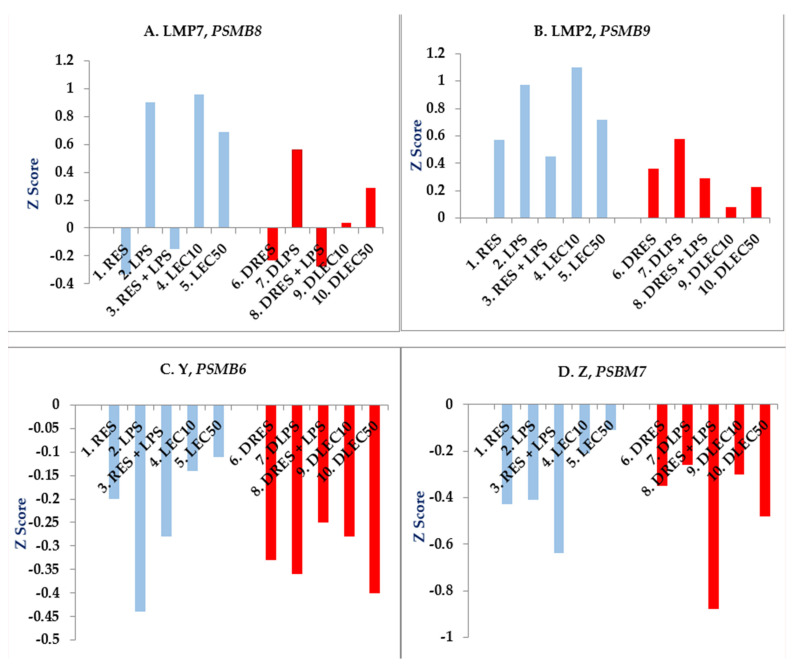
(**A**–**D**) Modulation of proteasome subunits: the effect of LPS, LEC10, LEC50, and RES on gene expression of proteasome’s subunits X, Y, Z, LMP7, LMP2, and LMP10 was determined in PBMCs from PwD2 and non-diabetic controls. Gene expression of subunits X and LMP10 was not induced in human PBMCs from non-diabetic controls and PwD2. Human PBMCs were treated with compounds and vehicle for 3h. RNA was extracted from the cells and analyzed using NGS. These data were extracted using the DEG analysis. Z score values were plotted against the proteasome subunits. Control PBMCs (C), RES (R); LPS (L); LPS + RES (L + R); LEC10; LEC50; and Diabetic PBMCs: RES (DR); LPS (DL); LPS + RES (DL + R); LEC10 (DLEC10); and LEC50 (DLEC50). Blue bars indicate values for gene expression of non-diabetic controls [54] and red bars indicate values for PwD2.

**LPS and LEC do not upregulate gene expression of proteasome’s proteases LMP7 (PSMB8) and LMP2 (PSMB9) in PBMCs from PwD2 to the same extent observed in non-diabetics. RES alone downregulates PSMB8 from PwD2 and controls (Figure 4A).** The modulation of most of these above-mentioned genes may be dependent on the UPS because its proteases degrade many of the short-lived proteins. To investigate major differences in proteasome subunits, PBMCs from LPS-treated controls and PwD2 were compared in Figure 4A–D.

We have previously shown that the proteasome is a central regulator of inflammation and cellular function by LPS. It contains the six protease subunits X, Y and Z, and the inducible subunits LMP7, LMP2, and LMP10. The PBMCs contain proteasome’s protease genes, predominantly subunits LMP7, LMP2, Y, and Z, while X and LMP10 were not significantly expressed in PBMCs (all six subunits are usually expressed in mouse C57Bl/6 macrophages and predominantly X, Y, and Z in RAW 264.7 cells). LPS and LEC50 upregulated the gene expression of *PSMB8* (LMP7) and *PSMB9* (LMP2) in non-diabetic PBMC, but to a lesser extent in those from PwD2 (Figure 4A,B), but *PSMB6* (Y) and *PSMB7* (Z) were downregulated in both controls and PwD2 (Figure 4C,D). RES downregulated the LPS-induced gene expression of *PSMB8* and *PSMB9* in PBMCs of both PwD2 and controls.

**Figure 5:**
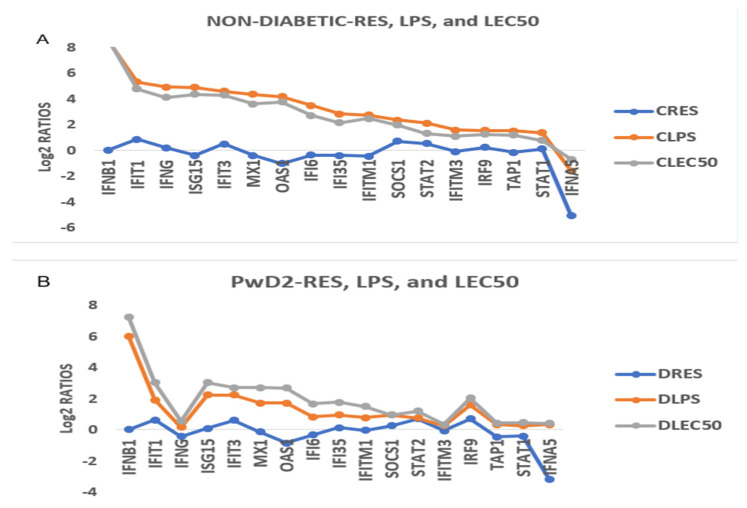
**IFN-induced genes** were downregulated in PBMCs from PwD2 as compared to non-diabetic controls in response to LPS, except for IFN-β. RES showed an inhibition or no change in gene expression, whereas LPS and LEC10 showed an upregulation of these genes. CRES, CLPS, and CLEC50 (controls) and DRES, DLPS, and DLEC50 (PwD2) were compared. (**A**) Non-diabetic control and (**B**) PwD2.

**LPS and LEC10 did not robustly upregulate gene expression of inflammatory IFN-γ- and IFN-λ-induced genes in PBMCs from PwD2 to the same extent as those from the non-diabetic controls.** We analyzed the data on the modulation of IFN-induced genes (Figure 5A,B). These genes included interferon β 1 (IFNB1), interferon-induced protein 1 (IFIT1), IFN-γ, interferon-stimulated gene 15 (ISG-15), IFIT3, interferon-induced antiviral protein (MX1), 2′-5′oligoadenylate synthetase1 (OAS1), IFI6, IFI35, interferon-induced membrane protein 1 (IFITM1), suppressor of cytokine signaling 1 (SOCS1), signal transducer and activator of transcription 2 (STAT2), IFITM3, interferon regulatory factor 9 (IRF9), the transporter associated with antigen processing1 (TAP1), STAT1, and interferon α5 (IFNA5).

Gene expression of mediators involved in IFN-β signaling pathways. Gene expression of IFN-β1 was similar in PBMCs from non-diabetic and PwD2 in response to LPS. IFIT1, IFN-γ, ISG-15, IFIT3, MX1, and OASI were robustly downregulated in non-diabetic controls and PwD2 with RES. RES showed a major downregulation in gene expression of IFN-α 5 (IFNA5), whereas LPS and LEC10 showed no upregulation of these genes. CRES, CLPS, and CLEC50 (controls) and DRES, DLPS, and DLEC50 (PwD2) were compared (Figure 5A,B).

**LEC50 alone upregulated gene expression of mediators involved in signaling pathways in PBMCs from PwD2.** The analysis of these data revealed that LEC50 alone could upregulate the expression of genes important in causing inflammation like LPS does in PBMCs from PwD2, with a few exceptions [54]. Several genes that were upregulated by LEC50 alone are listed in Table S5A. These inflammatory genes include *IL-6*, *IL-1A*, chemokines *CCL3*, *CXCL1*, and *CXCL3*; colony stimulating factor 3 (*CSF3*), coagulation factor (*F3*), chemokine ligand 4 (*CCL4*), *IL-1B*, *COX-2*, immune responsive gene 1 (*IRG1*), colony stimulating factor 2 (*CSF2*), *TNF*-α, Ras and Rab interactor 2 (*RIN2*), *IL-7* (interleukin 7, hematopoietic growth factor), *IFN-γ*, *USP18*, *CXCL11*, *RSAD2*, *IFI6*, *CACNA1A*, *FAM19A2*, *LAMP3*, *BCL2L14*, *SIGLEC1*, *ISG15*, and *CCNA1* (Cyclin A1) (Table S5A). The expression of more than 600 genes important in inflammation was modulated by LEC50 alone, but we have included only those whose *p* values were significant. *p* values < 4.98 × 10^−5^.

**LEC50 alone also showed less robust expression of genes of key mediators involved in several signaling pathways in PBMC from PwD2, as compared to non-diabetic controls.** Several important genes were also downregulated by LEC50, as listed in Table S5B. These genes included serine dehydratase (*SDS*, the deamination of L-serine to yield pyruvate), heme-oxygenase 1 (*HMOX1*, degrades heme to biliverdin and bilirubin), *CD163* (glycoprotein, is a sensor for Gram-negative and Gram-positive bacteria, scavenger receptor for hemoglobin-haptoglobin complex), fibrinogen (*FGL2* induces blood clots, but the deficiency causes bleeding and thrombosis), and chemokine receptor 1 (*CCR1*). Other downregulated genes include pyruvate dehydrogenase kinase isozyme 4 (*PDK4*), *CD14* (LPS binding protein), claudin 5 (*CLDN5*, integral membrane proteins and components of tight junction strands in endothelial cells), insulin receptor (*INSR* binds insulin), NRL family CARD domain (*NRLC4*), vitamin D3 receptor (*VDR*), N-acetylneuraminate pyruvate lyase (*NPL*, N-acetyl-D-mannosamine and pyruvate are products of this enzyme), and lysozyme (*LYZ*, antimicrobial enzyme). This suggests that LEC50 does not elicit robust upregulation of several genes that are important for Th1 immune response to infections in PBMC from PwD2, especially IFN-γ. Table S6 shows the modulation of transcription factor genes by various treatments of PBMCs from non-diabetic controls vs. PwD2.

**Figure 6:**
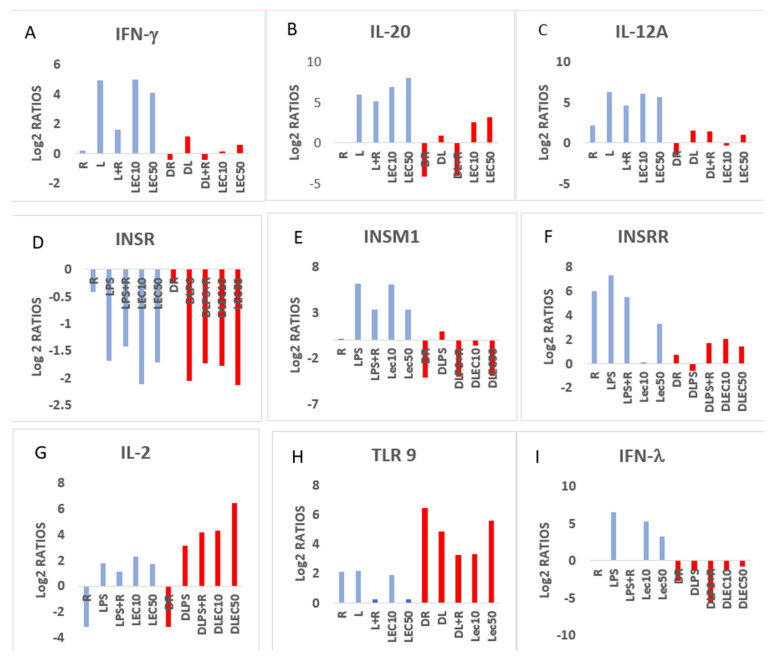
Cytokine and other inflammation-linked genes modulated by RES (R), LPS (L), LPS + RES (LPS + R, LR), LEC10 (lectin 10 μg/mL) and LEC50 (lectin 50 μg/mL) in PBMCs from non-diabetic controls (blue bars); and DRES (DR), DLPS (DL), DLPS + RES (DL + R), DLEC10 (lectin 10 μg/mL) and DLEC50 (lectin 50 μg/mL) in PBMCs from PwD2 (red bars). Cytokine genes are differentially regulated in PBMCs of non-diabetic controls and PwD2. (**A**). IFN-γ, (**B**). IL-20, (**C**). IL-12A, (**D**). INSR, (**E**). INSM1, (**F**). INSRR, (**G**). IL-2, (**H**). TLR9, and (**I**). IFN-λ.

**LEC50 downregulates gene expression of mediators involved in four major pathways, and these include: PPAR signaling, antioxidant action of vitamin C, LXR/RXR activation (Figure 2), and Gαι signaling.** LPS induces IFN-γ, IL-4 in PBMCs in non-diabetic controls (CLPS), but to a lesser extent in PwD2 (DLPS). Importantly, soybean LEC50 upregulated the expression of many of the genes that toxic LPS does in non-diabetic controls [54] (Figure 6, CLEC10, CLEC50, blue bars), but not to that extent in PwD2 (LEC10, LEC50, red bars). However, LEC10 and LEC50 can reverse this effect by inducing the gene expression of some of these cytokines in PBMCs from PwD2. Several other cytokines were modulated differentially, as shown in Table 2, Figure 6.

**Figure 7:**
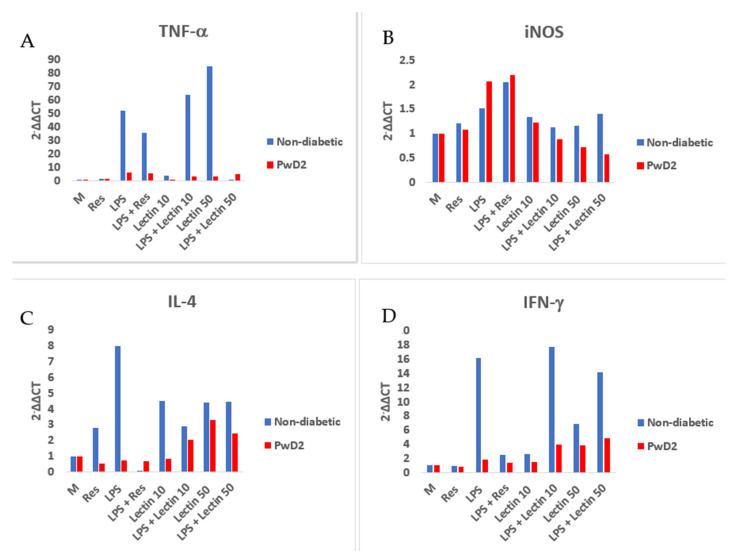
RNA was extracted from cells and gene expression was analyzed using qRT-PCR for validation of NGS data (Expt. 5). (**A**,**B**) TNF-α and iNOS genes modulated by medium control, LEC10 and LEC50, (**C**,**D**) IL-4 and IFN-γ genes modulated by different treatments in PBMCs of non-diabetics and PwD2.

**Figure 8:**
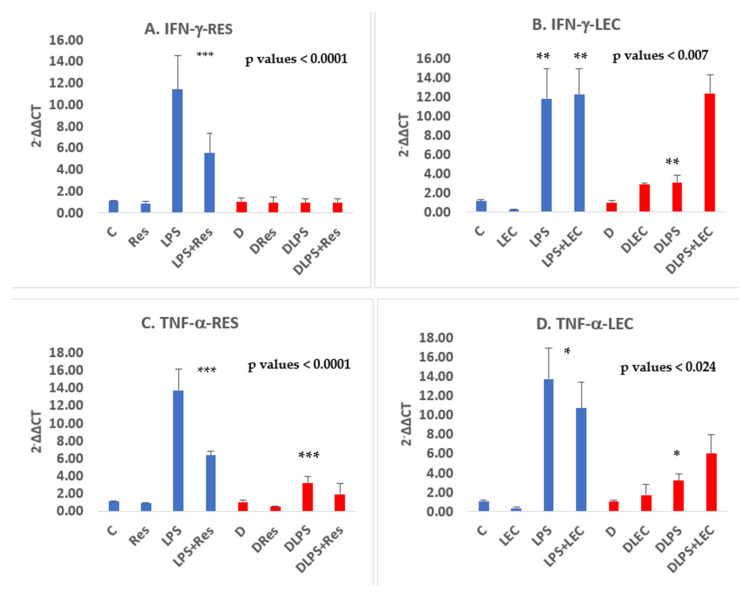
PBMCs were treated with either vehicle, RES, LPS, LPS + RES, or LEC10. RNA was extracted from cells and gene expression of IFN-γ and TNF-α was analyzed using qRT-PCR. Mean ± SEM. In these graphs, C, non-diabetic control was used to calculate 2^−ΔΔCT^ for non-diabetics, and D control was used for diabetics. LPS did not induce gene expression of IFN-γ as robustly in PBMCs from PwD2, compared to non-diabetic controls. Values in a column sharing a common asterisk with proteasome modulators were significantly different at *, **, *** *p* < 0.024, 0.007, 0.0001, using 1-way Anova. 2^−ΔΔCT^ = Relative quantification RQ. (Experiments 1–4 are described in the Section 4).

The NGS data for the gene expression for two cytokines, *TNF-α* and *IFN-γ*, was validated by qPCR using PBMC from non-diabetic vs. PwD2 in four additional experiments and the results were like those obtained in this study (Figure 7). The data and the trend with proteasome modulators were reproducible. A minimal upregulation of gene expression of *TNF-α* and *IFN-γ* was observed. LEC upregulated the gene expression of TNF-α and IFN-γ dose-dependently in the PBMCs of non-diabetics, but not in those from PwD2 (Figure 7 and Figure 8). Moreover, LEC10 upregulated the gene expression of IL-2 more robustly in PBMCs from PwD2, as compared to IFN-γ (Figure 7). The gene expression of iNOS and TNF-α was downregulated in PBMCs from PwD2, as compared to non-diabetic controls, as shown in Figure 8.

**Figure 9:**
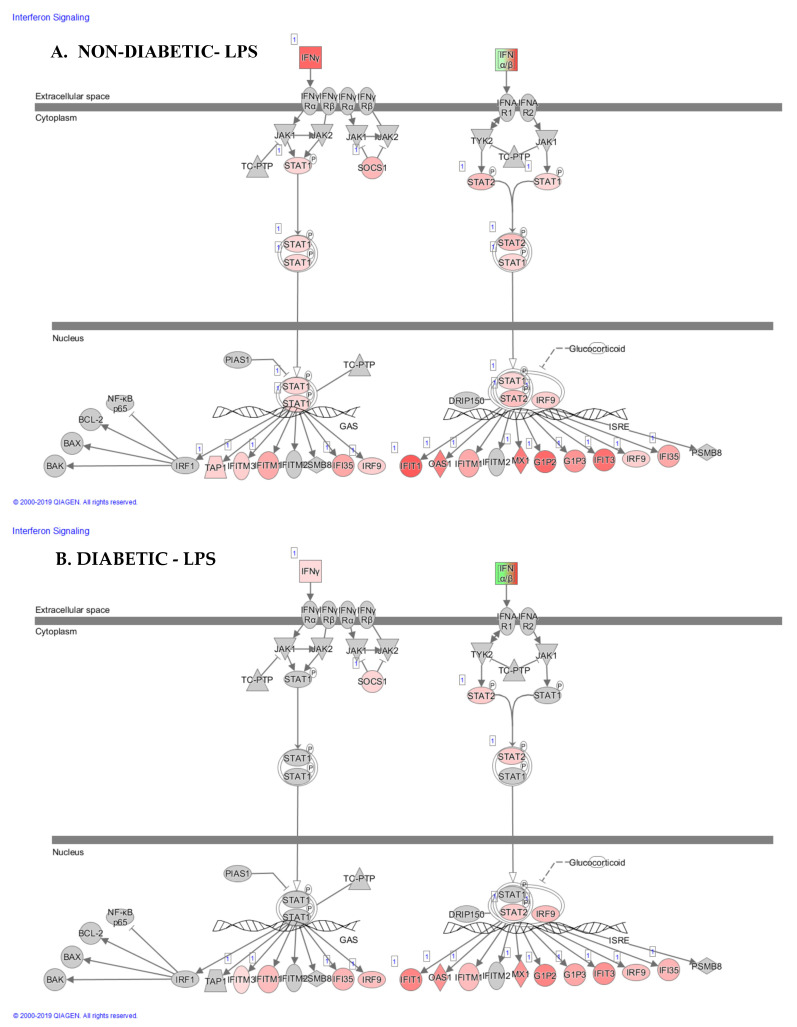
The interferon signaling pathway in human PBMC. The interferon signaling pathway plays a critical role in the induction of immune response by LPS or viruses: the STAT1 and STAT2 transcription factors play a major role in the transcription of interferon-activated genes. (**A**). LPS-non-diabetic and (**B**). LPS-diabetic. Red color denotes activation of genes, green color denotes repression of genes, and grey color denotes no change in gene expression.

**Gene expression of LPS-induced STAT1 and IFN-γ was not robustly upregulated in PBMCs from PwD2 as compared to those from non-diabetic controls.** PBMCs from non-diabetic controls show a robust upregulation of the gene expression response to LPS. Gene expression levels of IFN-γ, STAT1, STAT2, and many of the downstream proteins were upregulated with LPS in non-diabetic controls (Figure 9A) [54]. In contrast, PBMCs from PwD2 failed to elicit a robust gene expression response to LPS, and a significant upregulation in the gene expression of IFN-γ signaling pathways via STAT1 (Figure 9B).

## 3. Discussion

Mounting a good immune response (upregulation of IFN-γ) to viral or bacterial toxins is essential for the proper disposal of infectious agents, as observed in PBMCs from non-diabetic controls [54]. Proteasomes play a central role in most of the functions of the cell via the degradation of proteins. Proteasomes utilize their six proteases to degrade unwanted proteins in the cell, signaling proteins, transcription factors, cell-cycle proteins, cytokines, and to control the cellular metabolism at the proper time. Recently, we reported that proteasome activators, LPS and LEC, upregulate LMP7 and several inflammatory mediators, while proteasome inhibitor RES downregulates some of these mediators in PBMCs from non-diabetic controls. Therefore, we wanted to determine if chronically inflamed PBMCs from PwD2 would also react similarly to proteasome modulators, such as LPS, or other toxins, such as soybean LEC and flavonoids (RES), as efficiently as those from non-diabetic controls. Unexpected results were obtained since PBMCs from PwD2 showed a failure to elicit a normal response to LPS and lectins. LPS could not induce the gene expression of IL-12A, HLA complex II, IFN-γ, TNF-α, LMP7, and IFN-*λ* presumably because PBMCs from PwD2 were already in a more inflamed and insulin-resistant state. This may lead the PBMCs to become tolerant and refractory to LPS as discussed below. We also provide evidence here that dietary components, such as RES (proteasome inhibitor of predominantly subunit LMP7), showed a comparable anti-inflammatory response in the expression of some genes, while LEC showed a normal or reduced induction of gene expression response in PBMCs from PwD2, as compared to non-diabetic controls.

Our data showed that in ex vivo culture, PBMCs from PwD2 were dysregulated immunologically since gene expression of mediators of several pathways and cytokines were affected. These PBMCs were relatively unresponsive to LPS with respect to the gene expression of IFN-γ and key mediators in signaling pathways as compared to non-diabetic controls. IFN-γ belongs to a family of cytokines that function as potent macrophage-activating, microbicidal, and antiviral agents in upregulating host defense mechanisms. Major differences were noted in the DEG analysis of RNA from PBMCs of non-diabetic controls vs. PwD2, only upon further treatment with LPS or LEC.

This manuscript provides strong evidence to support the conclusion that PBMCs from PwD2 show a failure in eliciting a normal response to LPS, as compared to non-diabetic controls. Importantly, this failure in eliciting an upregulation in the gene expression of key mediators in IFN-γ signaling and others (Figure 6), such as interferon lambda (*IFN-λ*, involved with dendritic cell function), transporter associated with antigen presentation (*TAP1*), tumor necrosis factor (*TNF-α*), signal-transducer and activator of transcription-1 (*STAT1*), cytotoxic T-lymphocyte-associated protein 4 (*CTLA-4*), also known as CD152 (functions as immune checkpoint protein). Other genes include immune responses of T cells in cancer cells, insulin-receptor-related protein (*INSRR*), MHC II complex *HLA-DQB* and *HLA-DQA*, *IL-12A*, *IL-20*, glucose transporter 4 (*GLUT4*), insulinoma-associated protein 1 (*INSM1*), and insulin receptor (*INSR*). The gene expression values of some of these mediators are shown in Table 2.

In contrast, some genes were actually upregulated by LPS and LEC50 in PBMCs from PwD2, and these included *IL-2* (IL-2 is known to also activate CD8 and B cells directly, without the activation of MHC-I molecules) [55], *TLR9* (receptor for methylated DNA), *CLEC6A* (C-type lectin 6A), *CDKN2B-AS1* (long non-coding RNA involved in cancer and other malignancies), *CDC42-IT1* (cell cycle, Rho family of GTPases), *HLA-J* (MHC-I), and *BCL2L14* (anti-apoptotic factor), as compared to non-diabetic controls. There was upregulation in gene expression of *IL-12B*, *IL-1β*, *IL-6*, *IFN-β1*, *HDAC9*, *F3* (tissue factor), and *F8* (coagulation pathway causes blood clots) in PBMCs of both non-diabetic controls and PwD2.

There are several reasons why PBMCs from PwD2 might have failed to elicit a robust immune response to LPS and LEC with respect to IFN-γ signaling and other pathways. This could be due to some or all of the following factors: (1) Metformin is an anti-inflammatory compound and is widely used for PwD2 [56,57,58]; (2) The low induction of the gene expression of LMP7 (*PSMB8)*, and other subunits of the proteasome; (3) The induction of genes linked to epigenetic pathways; (4) The weak dendritic–T cell interaction; (5) Tolerance due to previous exposure to LPS or lectins, or T-cell senescence due to chronic inflammation; (6) Tolerant PBMCs being in a state where IFN-γ is no longer inducible. Therefore, significant upregulation in the gene expression of the IFN-γ signaling pathways via STAT1 was not observed. Previously, some researchers have reported a slight upregulation of IFN-γ, while others have reported a downregulation in PwD2 [59,60,61,62]. We have shown for the first time, that PBMCs from PwD2 show a dysregulated response to toxins such as LPS and lectins, as described below. Moreover, the repression of several genes was observed in PBMCs from PwD2, which could be due to lower gene expression of two important transcription factors that were downregulated, *T-box 3* (TBX3) and neuregulin 1 (*NRG*) (Table S6). These transcription factors have previously been shown to be important for cancer and obesity, respectively [63,64]. The net result observed in PBMCs from PwD2 could be due to the weak crosstalk between the dendritic cells (low abundance of *CD80* and *MHC-II*) and T cells.

The upregulated genes in PBMCs of PwD2 (without LPS treatment) included enzymes, such as the mitochondrial isozyme of pyruvate dehydrogenase kinase 4 (*PDK4*), the increased expression of which is linked to decreased metabolism, hypoxia, conservation of glucose by decreasing acetyl-CoA, (which enters in the citric acid cycle, and produces ATP). ATP is required for proteasomal activation and inflammatory processes because protein degradation by the UPS would shut down without it. It is well established that LPS causes insulin resistance in the mouse, as well as in the human model of DM2 [65,66,67,68]. LPS/LEC causes the downregulation of gene expression of insulin receptor (*INSR*) and the upregulation of *INSRR*, *INSM1* [69], and *TRIP10* in PBMCs from PwD2 and healthy controls, lectins may also be contributing to hormonal changes, such as insulin resistance and thyroid hormone receptor interaction, resulting in metabolic changes in the host.

**Figure 10:**
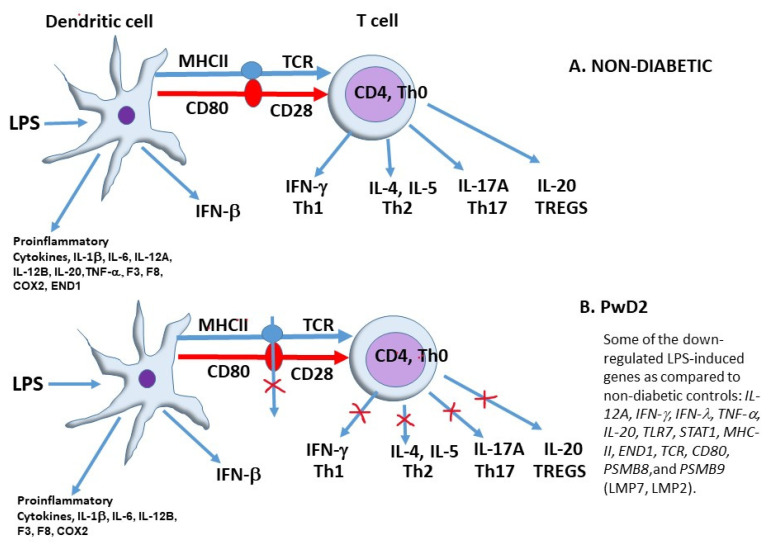
A simplistic model for LPS-induced genes in non-diabetic vs. PwD2. Figure shows the modulation of gene expression in treated PBMCs of PwD2/non-diabetics with RES, LPS, and LEC. Respective controls were used for analyses. A simplistic model for activation of innate and acquired immunity in PBMCs based on LPS-mediated gene expression. The role of RES, LPS, LPS + RES, and LEC10 on the innate and acquired immunity in human PBMCs from non-diabetic controls and PwD2 was determined.

To place these observations within the framework of what is currently understood regarding LPS-mediated signaling pathways, we propose the following simplistic model for mechanisms involved in human PBMC in PwD2 and non-diabetics in Figure 10. It is well-established that dendritic cells can cross talk with the naïve Th0 CD4 cells using their MHC-II molecules and T cell receptor (TCR) via interaction of CD28 and CD80 (B7). LPS leads to the induction of the gene expression of pro-inflammatory cytokines (cytokine storm) and mediators such as *IL-1β*, *IL-6*, *IL-12*, *TNF-α*, *IFN-β*, *F3*, *F8*, *COX2*, and *IL-27* in PBMCs. The IFN-γ (from Th1 or natural killer cells, NK cells) induced by agonists and transcription factor STAT1 activate LMP7 subunits of the proteasomes of Th1 cells and macrophages [54]. While Th2 cytokines IL-2, IL-4, IL-5, STAT6, and GATA3 activate B cells (CD19) to induce memory cells and plasma cell antibodies (IgM, IgG, IgA and IgE) in non-diabetic cells. We report for the first time that LPS and LEC50 treatments lead to a robust induction of gene expression of pro-inflammatory cytokines and innate immunity mediators such as *IL-1β*, *IL-6*, *IL-12B*, *IFN-β*, *F3*, *F8*, *COX2*, and *IL-27* in PBMCs from non-diabetic controls and PwD2 (using NGS). In contrast, the gene expression of *IL-12A*, *STAT1*, *PSMB8*, and *MHC-II HLA DQB*, as well as most IFN-γ-induced proteins and several other key cytokine genes involved in the adaptive immune response observed in non-diabetics, were not upregulated to the same extent in PBMCs of PwD2. PBMCs from PwD2 upon activation with LPS showed minimal gene expression of T cell cytokines with respect to Th1 (*IFN-γ*), Th2 (*IL-4*, *IL-5*), Th17 (*IL-17*) and Treg cell (*IL-10, IL-20*) cytokines, except for Th1 (*IL-2)*. IL-2 is known to activate Treg cells, which calms down the inflammatory response [55].

The natural products RES reduced the gene expression of these IFN-γ-signaling cytokine gene response in T cells even further; in contrast, LEC50 showed activation of the same inflammatory gene expression patterns as LPS in PBMCs of controls and PwD2. LEC could not upregulate the gene expression of *IFN-γ, PSMB8, CD80, TCR*, and *MHC II* involved in adaptive immune response and T-cell killing to the same extent in PBMCs of PwD2, as compared to non-diabetic controls. These data could explain why PwD2 are more vulnerable to bacterial and viral diseases as compared to non-diabetic individuals. These data also provide support for the hypothesis that bacterial and plant toxins (LPS/bacteria/viruses and lectins from plants) would react differentially to elicit the gene upregulation of important mediators in PBMCs from non-diabetic controls and PwD2.

This study also revealed for the first time that bacterial (LPS) and plant toxins may cause robust inflammation in healthy controls and PwD2, leading to the downregulation of the gene expression of the insulin receptor (*INSR).* In addition, the gene expression of INSRR and INSM1 in toxin treated PBMCs from PwD2 was upregulated to a lesser extent than non-diabetics, which may lead to insulin resistance and problems with cell maturity in the host. LPS is known to circulate in the bloodstream, adipose tissue, and the liver, to induce insulin resistance in vivo, but this has not been previously shown with LEC [70,71]. This study reports that PBMCs from people of the same age and weight respond differentially to LPS and LEC, depending on whether they were non-diabetic or PwD2. LPS and LEC also robustly activated the thyroid hormone receptor-interacting protein 10 (*TRIP10*, which can change the metabolic response) in both PBMCs, from non-diabetics and PwD2. The enzymes responsible for the biosynthesis of thyroid hormones are degraded by the proteasomes. Proteasome modulators RES inhibits LMP7 and reduces inflammation, while LEC (activates LMP7 and induces inflammation). Recently, we have shown that a mixture of RES, vitamin D3 and δ-tocotrienols (NS-3) as food supplements reduce blood sugar and inflammatory cytokines in PwD2 in a clinical study in vivo [72]. More genomic, proteomic research, and clinical studies using proteasome modulators in humans are, therefore, required to fully understand how various dietary components, such as lectins/flavonoids [73], affect lectin receptors, transcription factors, and proteasomes in DM2.

## 4. Materials and Methods

### 4.1. Experiments Prior to RNAseq Analysis

To gain a comprehensive picture for the contribution of dietary nutrients we had a total of *n* = 5 non-diabetic controls and *n* = 5 PwD2 (Table 3). The first four sets were analyzed using selected primers by RT-PCR to find the proper dose of modulators. The fifth sample with 16 treatments was analyzed by Novogene using next generation sequencing (NGS) using the Illumina supports, as described below and in reference [54].

### 4.2. Reagents

Deep rough chemotype LPS (Re-LPS) from *E. coli* D31m4 was purified as described by Qureshi et al. [74,75,76]. For tissue culture studies, RPMI 1640 Medium, heat-inactivated low-endotoxin fetal bovine serum (FBS), and gentamycin were purchased from Cambrex (Walkersville, MD, USA). RNeasy one-step kit was purchased from QIAGEN sciences (Germantown, MD, USA). Highly purified (affinity column purified and stated by the company to have undetectable LPS, soybean lectin LEC from Glycine max) was purchased from Sigma-Aldrich (St. Louis, MO, USA). Trans-resveratrol (RES > 99% HPLC pure) was also purchased from Sigma-Aldrich (St. Louis, MO, USA). PBMCs of a normal healthy individual and PwD2 were purchased from STEMCELL technologies (Vancouver, BC, Canada) Technologies or from Precision for Medicine (Frederick, MD, USA).

### 4.3. Detection of Cell Viability and Isolation of Total Cellular RNA

The viability and number of PBMCs were determined by the trypan blue dye exclusion test and by using a cell counter. After treatment, cells were washed with PBS and total RNA was extracted by using RNeasy mini kit (Qiagen, Germantown, MD, USA) as per the manufacturer’s instructions.

### 4.4. Experiments Prior to RNAseq Analysis

Viable PBMCs (5 × 10^6^/10 mL/plate, duplicates) were treated with: 1. medium only; 2. RES (80 μM); 3. LPS (10 ng/mL); 4. LPS 10 ng/mL+ RES 80 μM; 5. LEC10 (10 μg/mL); 6. LPS 10 ng/mL + LEC10 μg; 7. LEC 50 (50 μg/mL); 8. LPS 10 ng/mL + LEC50 (50 μg/mL) for 3 h for the non-diabetic. Reference 54 used the same treatments for PBMCs from controls, as shown in the Table 4 below. All samples were adjusted to contain the same final concentration of DMSO (0.2%). At 3 h of treatment, total RNA was extracted from treated / untreated PBMCs from non-diabetic controls and from PwD2 and purified further using an affinity resin column (RNeasy, Qiagen, Chatsworth, CA, USA) [54].

### 4.5. Total Cellular RNA Isolation and qPCR

qPCR was performed with total RNA isolated from cells treated with RES, LEC and/or LPS, as described previously [28,29,54]. Total cellular RNA was isolated with a RNeasy mini kit according to the manufacturer’s instructions (RNeasy, Qiagen, Chatsworth, CA, USA). To check the purity of the RNA for cytokines, reverse transcription and PCR were conducted using one step qPCR Kit (Qiagen, Chatsworth, CA), according to the manufacturer’s instruction.

### 4.6. Sample Preparation for RNAseq Analysis

Five to 8 μg of total RNA from each sample were provided to Novogene Global (Sacramento, CA, USA) for RNAseq analyses using the human Illumina system (Santa Clara, CA, USA), as described previously [54]. The purity of total RNA was assessed using the following tests, Nanodrop (OD 260/280), agarose gel electrophoresis and Agilent 2100 analysis to check RNA integrity. The following procedures were carried out by Novogene: mRNA enrichment, conversion to double-stranded cDNA, end repair, poly-A adaptor addition, fragment selection and PCR, library quality assessment, and Illumina sequencing. An average of 44–61 million raw read counts were obtained. Novogene used the STAR software for alignment for RNA-seq data analysis. Read counts are proportional to gene expression levels, gene length, and sequencing depth. The differential gene analysis was carried out on two samples (control vs. treatment group) at a time using the DESeq2 R package. The threshold of differential expression genes is log2 fold change >1, and *p* value < 0.05 [54].

### 4.7. Data Analysis, Network and Pathway Analysis

Gene expression data were first imported in the differentially expressed genes (DEG) program and numbers were corrected for differences in the IIlumina analysis. The numbers presented in Tables S1–S6 are averages from 2 incubations for each treatment; the log ratio values were normalized to a scale of 0 (instead of 1, which shows decimals), the expression values of upregulated genes showed positive numbers, and the downregulated ones showed negative numbers (called normalized ratios, a log ratio of 2 is equivalent to a fold change of 4); these ratios were imported into the ingenuity pathways analysis (IPA) software (Ingenuity Systems, Mountain View, CA, USA) [28,29,54]. This is a web-based tool that is predicated on more than 200,000 full-text articles and has information based upon 7900 human and mouse genes. This system categorizes genes into high-level cellular functions and canonical pathways and has been used to characterize genes important in human systemic inflammation [28,29,54]. Genes found to be significantly activated were categorized based on different pathways and networks available in the database and ranked by score, as described previously [28,29,54]. Genes identified as statistically different from the background, in terms of activation relative to control cells, were analyzed and mapped into different pathways.

### 4.8. Statistical Analysis

The data were analyzed using analysis of covariance (ANOVA) to compare means of pre-treatment versus post-treatment. Data are reported as mean + SD (standard deviation in Figure 8. The statistical significance level was set at 5% (*p* < 0.05) [54].

## 5. Conclusions

These findings provide insights into immunologic mechanisms operative in PwD2 in the presence of toxins. Collectively, these novel findings strongly support the existence of a highly dysregulated level of immune activity in PBMCs from PwD2 relative to non-diabetic adults. In PBMCs from non-diabetics, LPS induces gene expression of several cytokines. LPS induces gene expression of IL-12A in monocytes and macrophages, leading to the activation of IFN-γ from NK or Th1 cells. Th1 cells and dendritic cells signal via the interaction of CD80 with CD28, and MHC II molecules with T-cell receptor. IFN-γ amplifies the entire signaling pathway by upregulating LMP7 (*PSMB8*), LMP2 (*PSMB9*) and several induced proteins in PBMCs. The continued interaction of cells with LPS and IFN-g leads to tolerance, where there is no significant change in the gene expression of *PSMB8* or other subunits of the proteasome with agonists. However, in PBMCs from PwD2 this regulation is lost because the cells do not robustly induce the gene expression of *PSMB8* with LPS, and there is a weak interaction between the dendritic cells and Th1 cells. Therefore, induction of gene expression of IL-12A, IFN-γ, STAT1, MHC-II molecules, LMP7, and several IFN-γ-induced genes was not robustly elicited in response to LPS in PBMCs from PwD2, as compared to those from non-diabetics (Figure 10). RES (with/without LPS) downregulates the gene expression of *PSMB8* and the mediators of IFN-γ pathway even further, while LEC upregulates the gene expression of these mediators. The data presented in this manuscript would be very useful in designing a therapeutic approach for most inflammatory and infectious diseases, such as diabetes, sepsis, tuberculosis, glaucoma, macular degeneration, neuroinflammatory disorders, COVID-19, and AIDS.

## Figures and Tables

**Table 1:** Differentially expressed gene (DEG) analysis modulated by RES, LPS, LPS + RES, LEC10, LEC10 + LPS, LEC50, and LEC50 + LPS in PBMCs from non-diabetic (N) and PwD2 (D) using next generation sequencing (NGS). Respective controls for non-diabetics and PwD2, C1, and C9 were used as controls, respectively.

**#**	**Treatments**	**Total DEGS**	**Upregulated**	**Downregulated**
1	Control non-diabetics			
2	Resveratrol (RES)	26	4	22
3	Lipopolysaccharide (LPS)	680	555	125
4	LPS + RES	424	290	133
5	Lectin10 (LEC10)	295	207	28
6	LEC10 + LPS	640	534	106
7	LEC50	570	492	78
8	LEC50 + LPS	698	588	110
	**Treatments**	**Total DEGS**	**Upregulated**	**Downregulated**
9	Control PwD2 *			
10	Diabetes-Resveratrol (DRES)	40	14	26
11	Diabetes-Lipopolysaccharide (DLPS)	472	377	95
12	DLPS + RES	426	275	151
13	Diabetes-Lectin10 (DLEC10)	287	249	38
14	DLEC10 + LPS	469	382	87
15	Diabetes-Lectin 50 (DLEC50)	489	384	105
16	DLEC50 + LPS	494	383	111

* PwD2 = People with type 2 diabetes. DEGS = Differentially expressed genes. Blue color is used for gene expression for PBMCs from non-diabetic subjects and red color is used for PBMCs from PwD2 treated with different compounds.

**Table 2:** A summary of gene expression (Log2-fold values) of selected cytokines and other key mediators of signaling pathways. RNA was extracted from PBMCs from PwD2 (D), and non-diabetic controls (C) after treatment with RES (CR, DR), LPS (CL and DL), RES + LPS (CRL and DLR), LEC10 (CLEC10 and DLEC10), and values were analyzed using RNAseq (NGS) followed by IPA. Downregulated genes are boldfaced.

	CR	DR	CL	DL	CLR	DLR	CLEC10	DLEC10
IL6	**−0.095**	0.75	9.67	9.729	10.139	10.38	9.724	9.691
IL1B	**−0.956**	**−1.096**	7.304	6.026	7.467	6.401	7.38	6.32
PTGS2	**−0.541**	**−0.338**	7.018	6.09	7.396	6.733	7.012	6.349
TNF	**−0.043**	0.074	5.790	3.108	5.392	3.488	5.986	3.38
CXCL10	0.873	**−0.303**	4.645	1.922	2.06	**−0.226**	4.560	1.245
IFNG	0.190	**−0.453**	4.913	1.123	1.586	**−0.45**	4.953	0.144
CXCL8	**−0.893**	**−0.267**	3.913	2.696	3.845	2.894	3.974	2.832
IL12B			9.513	9.276	8.337	8.162	9.761	7.834
HMOX1	0.321	0.42	**−3.322**	**−3.731**	**−2.866**	**−3.902**	**−3.399**	**−3.252**
IL12A	2.13	**−1.496**	6.216	1.556	4.646	1.435	6.08	**−0.314**
NLRP3	0.361	0.315	3.019	2.53	4.083	3.449	3.124	3.126
TLR7	**−0.116**	**−0.294**	3.156	2.313	1.854	1.369	3.089	1.665
SOD2	0.783	0.839	2.787	2.462	2.704	2.335	2.821	2.321
CD80	0.936	**−0.139**	3.120	1.125	1.541	0.629	3.183	1.631
CD40	0.383	0.422	1.284	0.61	1.495	1.232	1.188	0.274
SLC1A2	**−0.23**	**−1.222**	3.167	2.706	0.523	0.084	2.843	1.875
IFNB1			8.443	6.524	7.453	7.167	8.194	6.013
IRF7	0.178	0.573	2.492	1.989	1.519	1.083	2.556	1.112
HLA-DOB	1.360	0.887	3.122	1.723	2.148	0.853	3.206	0.606
TGFB2	1.636	**−1.054**	3.349	0.607	1.609	**−0.374**	3.377	1.654
NFKB1	**−0.063**	**−0.142**	1.412	1.211	1.458	1.198	1.449	1.145
FAS	**−0.392**	**−0.555**	1.405	0.9	0.739	0.26	1.473	0.441
IRAK2	0.039	0.158	1.358	1.127	1.015	0.677	1.453	1.165
STAT1	0.09	**−0.429**	1.356	0.897	**−0.206**	**−0.656**	1.249	0.245
TICAM1	**−0.432**	**−0.352**	1.335	0.986	0.433	0.098	1.339	0.695
PLA2G4A	**−1.374**	**−1.702**	1.317	0.145	0.181	**−0.109**	1.088	**−0.101**
ZBTB12	1.68	0.759	2.26	1.236	2.055	1.81	1.262	1.175
IL1R1	**−0.904**	**−1.196**	1.184	0.76	0.095	**−0.138**	1.17	0.242
ACVR1C	0.034	**−1.107**	1.495	0.388	0.464	**−0.167**	1.303	0.457
JAK3	0.123	0.108	1.118	0.888	0.904	0.883	1.131	0.779
TGFB3	**−0.614**	**−0.287**	1.424	0.306	0.052	−0.159	0.377	0.206
GLS	0.31	0.185	1.060	0.597	0.688	0.583	0.893	0.422
MYD88	**−0.249**	**−0.367**	1.05	0.412	**−0.134**	**−0.413**	0.975	0.282
FASLG	**−0.542**	**−1.166**	1.085	0.461	0.076	**−0.813**	1.355	**−0.275**
RIPK1	0.128	0.08	1.031	0.566	0.485	0.413	1.012	0.156
PIK3R3	**−0.266**	**−1.235**	1.331	0.261	**−1.642**	**−2.063**	1.167	**−0.67**
CTLA4	**−0.119**	**−0.436**	1.171	0.604	0.451	**−0.204**	1.181	0.651
END1	1.4	**−0.547**	4.901	2.506	1.302	1.176	4.967	2.248
ATG7	**−0.38**	**−0.202**	1.167	1.374	0.767	1.184	1.228	1.310
TRIP10	**−0.192**	0.239	2.73	3.317	2.621	3.132	2.741	2.721
PSMB8	**−0.325**	**−0.147**	0.903	0.385	**−0.154**	**−0.424**	0.964	0.415
PSMB9	**0.574**	0.084	1.097	0.437	0.446	0.673	1.103	0.378

Abbreviations: Interleukin 6 (*IL6*), Interleukin 1β (*IL-1B*), PTGX2 (COX2), Tumor necrosis factor (TNF-α), CXCL10 (IP10), Interferon γ (IFN-γ), CXC Chemokine ligand 8. *CXCL8*, Interleukin IL-12β (*IL12B*), heme oxygenase 1 (*HMOX1*), Colony stimulating factor 1 receptor (*CSF1R*), Nod like receptors (*NLRP3*), cAMP responsive element binding protein (*CREB5*), Toll-like receptor 7 (*TLR7*), Superoxide dismutase 2 (*SOD2*), Cluster of differentiation 80, 40, (*CD80, CD40*), solute carrier family 1 member 2 (*SLC1 A2*), Interferon-β1 (*IFN-β1*), Major histocompatibility antigen class II DOB (*HLA DOB*), Caspase 8 and FADD like apoptosis regulator (*CFLAR*), Intercellular adhesion molecule1 (*ICAM1*), Purinergic receptor P2X7 (*P2RX7*), Transforming growth factor β2 (*TGFB2*), NRL apoptosis inhibitory protein (*NAIP*), TNF receptor family member 1A (*TNFRSF1A*), Nuclear factor of activated T cells 5 (*NFAT5*), Nuclear factor of kappa B subunit 1 P50, (*NFKB1*), FAS cell surface death receptor (*FAS*), Interleukin receptor associated kinase 2 (*IRAK2*), Signal transducer and activator of transcription 1 (*STAT1*), TIR domain containing adaptor molecule 1 (*TICAM1*), Phospholipase A2 group 4A (*PLA2G4A*), Zinc finger and BTB containing domain 12 (*ZBTB12*), Activin A receptor type 1 (*ACVR1C*), Janus kinase (*JAK3*), transforming growth factor B3 (*TGFB3*), Glutaminase (*GLS*), Innate immunity signal transducer adaptor (*MyD88*), FAS ligand (*FASLG*), Receptor interacting serine/threonine kinase 1 (*RIPK1*), phosphatidylinositol-4-phosphate 3-kinase catalytic subunit type 2 beta (*PIK3C2B*), PIK3R3, cytotoxic T-lymphocyte associated protein 4 (*CTLA4*), Endothelin 1 (*END1*), Autophagy linked gene 7 (*ATG7*) and Thyroid hormone receptor interactor protein-10 (*TRIP10*). Genes boldfaced were downregulated.

**Table 3:** PBMCs were chosen from PwD2, and non-diabetic controls as follows.

Expt 1	Sex	Age	Race	Weight	Drug/Metformin
PwD2 PBMCs	Male	49 yrs	Caucasian	90 kg	Yes
Non-diabetic control PBMCs	Male	27 yrs	African Amer.	112 kg	No
Expt 2					
PwD2 PBMCs	Female	52 yrs	Caucasian	110 kg	Yes
Non-diabetic control PBMCs	Female	49 yrs	Caucasian	63 kg	No
Expt 3					
PwD2 PBMCs	Male	49 yrs	Caucasian	90 kg	Yes
Non-diabetic control PBMCs	Male	54 yrs	Caucasian	105 kg	No
Expt 4					
PwD2 PBMCs	Female	52 yrs	Caucasian	110 kg	Yes
Non-diabetic control PBMCs	Female	48 yrs	Caucasian	106 kg	No
Expt 5					
PwD2 PBMCs	Male	65 yrs	Caucasian	108 kg	Yes
Non-diabetic control PBMCs	Male	60 yrs	Caucasian	92 kg	No

**Table 4:** Treatments for PBMCs from non-diabetic controls and PwD2.

1.	Control	9.	Control
2.	RES 80 µM	10.	RES 80 µM
3.	LPS 10 ng	11.	LPS 10 ng
4.	LPS + RES	12.	LPS + RES
5.	LEC10 µg	13.	LEC10 µg
6.	LEC10 + LPS	14.	LEC10 + LPS
7.	LEC50 µg	15.	LEC50 µg
8.	LEC50 + LPS	16.	LEC50 + LPS

## Data Availability

Data analyzed during this study are included in this article.

## Supplementary Materials

The following supporting information can be downloaded at: www.mdpi.com/article/10.3390/ijms24032732/s1.

## Abbreviations

Lipopolysaccharides (LPS), Resveratrol (RES), Soybean lectin (LEC), Ubiquitin proteasome system (UPS).
