# Supplementary material for: Dysregulation of Gene Expression of Key Signaling Mediators in PBMCs from People with Type 2 Diabetes Mellitus
Source: Int J Mol Sci. 2023 Feb 1;24(3):2732. doi: 10.3390/ijms24032732 (PMC9916932; doi:10.3390/ijms24032732)
Supplement: Supplementary file 1 [file ijms-24-02732-s001.zip › ijms-2109940-supplementary.pdf]

SUPPLEMENTARY MATERIAL.

TABLE S1A: Downregulated genes in PBMCs of PwD2 control/healthy control (PwD2C/HC). S1B: Upregulated genes in PBMCs of PwD2 control/healthy control (PwD2C/HC). (1A)			
**log2 Fold**	**p value**	**Gene Name**	**Gene Description**
-10.576	2.27x10-46	*HLA-DQB1*	**major_histocompatibility_complex_class_II_DQ_beta_1**
-5.4515	3.08 x10-25	*HLA-DQA1*	**major_histocompatibility_complex_class_II_DQ_alpha_1**
-6.9689	4.99 x10-16	*TBX3*	**T-box_3**
-3.7676	7.33 x10-15	*CDH6*	**cadherin_6_type_2_K-cadherin_(fetal_kidney)**
-4.0223	9.20 x10-15	*A4GALT*	**alpha_14-galactosyltransferase**
-3.5338	8.72 x10-12	*TMEM176A*	**transmembrane_protein_176A**
-3.0779	9.57 x10-12	*HIST1H1C*	**histone_cluster_1_H1c**
-8.5256	2.27 x10-09	*TPSAB1*	**tryptase_alpha/beta_1**
-2.8866	3.35 x10-09	*TMEM176B*	**transmembrane_protein_176B**
-5.621	4.11 x10-09	*AGAP7*	**ArfGAP_with_GTPase_domain_ankyrin_repeat_and_PH_domain_7**
-2.6061	4.79 x10-09	*FPR1*	**formyl_peptide_receptor_1**
-8.3521	1.51 x10-08	*PSPHP1*	**phosphoserine_phosphatase_pseudogene_1**
-2.7085	2.03 x10-08	*HSPA7*	**heat_shock_70kDa_protein_7_(HSP70B)**
-3.8739	2.95 x10-08	*FAM153A*	**family_with_sequence_similarity_153_member_A**
-2.6545	4.50 x10-08	*FCGR2C*	**Fc_fragment_of_IgG_low_affinity_IIc_receptor_for_(CD32)(gene/ps**
-2.5837	1.07 x10-07	*TRBV28*	**T_cell_receptor_beta_variable_28**
-3.1069	1.43 x10-07	*MLXIPL*	**MLX_interacting_protein-like**
-5.1977	2.51 x10-07	*KRT5*	**keratin_5**
-2.1726	4.09 x10-07	*RPS26*	**ribosomal_protein_S26**

-2.271	6.40 x10-07	*TMEM156*	**transmembrane_protein_156**
-2.1312	6.44 x10-07	*LYZ*	**lysozyme**
-2.7558	7.48 x10-07	*HIST1H2BD*	**histone_cluster_1_H2bd**
-2.9783	1.26 x10-06	*EMR4P*	**EGF-like_module_containing_mucin-like_hormone_receptor-like_4**
-2.9131	1.39 x10-06	*LINC00599*	**long_intergenic_non-protein_coding_RNA_599**
-2.4719	2.44 x10-06	*SCIMP*	**SLP_adaptor_and_CSK_interacting_membrane_protein**
-2.2572	4.73 x10-06	*PLD4*	**phospholipase_D_family_member_4**
-2.841	5.08 x10-06	*MPZL2*	**myelin_protein_zero-like_2**
-2.2099	5.08 x10-06	*CLEC10A*	**C-type_lectin_domain_family_10_member_A**
-7.5933	5.45 x10-06	*TUBB2B*	**tubulin_beta_2B_class_IIb**
-2.0777	5.60 x10-06	*MS4A6A*	**membrane-spanning_4-domains_subfamily_A_member_6A**
-2.6531	6.52 x10-06	*IGKV3-15*	**immunoglobulin_kappa_variable_3-15**
-2.1749	7.47 x10-06	*ATP8B4*	**ATPase_class_I_type_8B_member_4**
-2.7149	9.47 x10-06	*HIST1H1E*	**histone_cluster_1_H1e**
-1.8589	1.38 x10-05	*FCGR3A*	**Fc_fragment_of_IgG_low_affinity_IIIa_receptor_(CD16a)**
-1.9268	1.53 x10-05	*LGALS2*	**lectin_galactoside-binding_soluble_2**
-2.24	1.57 x10-05	*AMZ1*	**archaelysin_family_metallopeptidase_1**
-1.8877	2.22 x10-05	*AIF1*	**allograft_inflammatory_factor_1**
-1.9691	2.24 x10-05	*MS4A4A*	**membrane-spanning_4-domains_subfamily_A_member_4A**
-2.4143	2.30 x10-05	*BST1*	**bone_marrow_stromal_cell_antigen_1**
-2.0713	2.62 x10-05	*ITGB2-AS1*	**ITGB2_antisense_RNA_1**
-3.8948	2.88 x10-05	*S100B*	**S100_calcium_binding_protein_B**

-7.3192	3.05 x10-05	*ZNF223*	**zinc_finger_protein_223**
-3.5252	3.15 x10-05	*TRDV2*	**T_cell_receptor_delta_variable_2**
-1.8352	3.29 x10-05	*CD300LF*	**CD300_molecule-like_family_member_f**
-2.2756	3.71 x10-05	*HIST2H2AC*	**histone_cluster_2_H2ac**
-2.2836	4.01 x10-05	*SSTR3*	**somatostatin_receptor_3**
-3.8444	4.06 x10-05	*HIST1H1D*	**histone_cluster_1_H1d**
-3.8444	4.06 x10-05	*MMP7*	**matrix_metallopeptidase_7_(matrilysin_uterine)**
-7.2417	4.78 x10-05	*MYL4*	**myosin_light_chain_4_alkali; _atrial_embryonic**
-1.7586	5.08 x10-05	*IFITM3*	**interferon_induced_transmembrane_protein_3**
-1.8919	5.26 x10-05	*MNDA*	**myeloid_cell_nuclear_differentiation_antigen**
-2.7168	5.29 x10-05	*ASGR1*	**asialoglycoprotein_receptor_1**
-2.2546	5.91 x10-05	*CCDC144B*	**coiled-coil_domain_containing_144B_(pseudogene)**
-1.868	6.52 x10-05	*MRI1*	**methylthioribose-1-phosphate_isomerase_homolog_(S._cerevisiae)**
-7.1598	7.55 x10-05	*SLC44A5*	**solute_carrier_family_44_member_5**
-1.9124	8.76 x10-05	*TGM2*	**transglutaminase_2_(C_polypeptide_protein-glutamine-gamma-gluta**
-1.6797	0.000102	*IFITM10*	**interferon_induced_transmembrane_protein_10**
-1.7394	0.000105	*PLBD1*	**phospholipase_B_domain_containing_1**
-1.7091	0.000105	*DNAJC15*	**DnaJ_(Hsp40) _homolog_subfamily_C_member_15**
-2.2002	0.000106	*GGTA1P*	**glycoprotein_alpha-galactosyltransferase_1_pseudogene**
-1.6369	0.000109	*SLC12A7*	**solute_carrier_family_12_(potassium/chloride_transporters) _membe**
-4.3458	0.000110	*SIGLEC12*	**sialic_acid_binding_Ig-like_lectin_12_(gene/pseudogene)**
-1.8946	0.000115	*MS4A14*	**membrane-spanning_4-domains_subfamily_A_member_14**
-2.1332	0.000117	*MEG3*	**maternally_expressed_3_(non-protein_coding)**
-2.3213	0.000147	*CD1C*	**CD1c_molecule**
-1.6083	0.000157	*APOBEC3A*	**apolipoprotein_B_mRNA_editing_enzyme_catalytic_polypeptide-lik**
-1.6965	0.000162	*FPR2*	**formyl_peptide_receptor_2**

-2.0759	0.000181	***PROC***	**protein_C_(inactivator_of_coagulation_factors_Va_and_VIIIa)**
-6.9806	0.000194	***HIST1H1B***	**histone_cluster_1_H1b**
-1.8049	0.000208	***APCDD1L***	**adenomatosis_polyposis_coli_down-regulated_1-like**
-2.8554	0.000211	***DOC2GP***	**double_C2-like_domains_gamma_pseudogene**
-2.3277	0.000258	***OLFM2***	**olfactomedin_2**
-2.1371	0.000283	***APOL4***	**apolipoprotein_L_4**

(1B)

**Log2 Fold**	**p value**	**Gene Name**	**Gene Description**
5.1427	1.79×10^{-17}	***FAM21B***	family_with_sequence_similarity_21_member_B
3.7463	9.04×10^{-15}	***MDGA1***	MAM_domain_containing_glycosylphosphatidylinositol_anchor_1
5.1924	3.62×10^{-13}	***RGPD2***	RANBP2-like_and_GRIP_domain_containing_2
3.37	1.15×10^{-12}	***DDX11***	DEAD/H_(Asp-Glu-Ala-Asp/His)_box_helicase_11
4.7559	2.99×10^{-12}	***TM4SF1***	transmembrane_4_L_six_family_member_1
3.7743	7.76×10^{-12}	***CLDN5***	claudin_5
4.3439	3.81×10^{-11}	***HLA-K***	major_histocompatibility_complex_class_I_K_(pseudogene)
2.9694	4.84×10^{-11}	***CXCL1***	chemokine_(C-X-C_motif) _ligand_1_(melanoma_growth_stimulating
4.0477	2.23×10^{-10}	***NBPF13P***	neuroblastoma_breakpoint_family_member_13_pseudogene
8.6718	5.21×10^{-10}	***GSTM1***	glutathione_S-transferase_mu_1
2.8426	1.04×10^{-08}	***FMN1***	formin_1
2.5647	1.56×10^{-08}	***SPRY2***	sprouty_homolog_2_(Drosophila)
3.0059	1.97×10^{-08}	***NUAK1***	NUAK_family_SNF1-like_kinase_1
3.7928	1.12×10^{-07}	***DDX11L10***	DEAD/H_(Asp-Glu-Ala-Asp/His)_box_helicase_11_like_10
3.4371	1.74×10^{-07}	***TRGV5P***	T_cell_receptor_gamma_variable_5P_(pseudogene)
2.5123	1.77×10^{-07}	***EVC***	Ellis_van_Creveld_syndrome
3.5249	2.49×10^{-07}	***TRGV5***	T_cell_receptor_gamma_variable_5
2.8043	3.01×10^{-07}	***KRT1***	keratin_1
2.1912	3.13×10^{-07}	***THBS1***	thrombospondin_1

2.5195	4.00 x10-07	*NLRP6*	NLR_family_pyrin_domain_containing_6
2.1580	4.65 x10-07	*SERPINB2*	serpin_peptidase_inhibitor_clade_B_(ovalbumin)_member_2
2.5358	6.22 x10-07	*AKR1C1*	aldo-keto_reductase_family_1_member_C1
2.3683	7.80 x10-07	*GSTM3*	glutathione_S-transferase_mu_3_(brain)
3.0005	8.41 x10-07	*FLT4*	fms-related_tyrosine_kinase_4
2.8017	8.60 x10-07	*TREML1*	triggering_receptor_expressed_on_myeloid_cells-like_1
4.2248	8.61 x10-07	*MYL9*	myosin_light_chain_9_regulatory
3.2449	9.44 x10-07	*ELOVL4*	ELOVL_fatty_acid_elongase_4
2.1546	1.56 x10-06	*PCSK1N*	proprotein_convertase_subtilisin/kexin_type_1_inhibitor
2.4076	1.79 x10-06	*GFPT2*	glutamine-fructose-6-phosphate_transaminase_2
4.765	2.59 x10-06	*TGM3*	transglutaminase_3_(E_polypeptide_protein-glutamine-gamma-glutam
3.0292	2.96 x10-06	*AKR1C2*	aldo-keto_reductase_family_1_member_C2
7.648	3.61 x10-06	*HLA-DRB6*	major_histocompatibility_complex_class_II_DR_beta_6_(pseudogene)
2.2451	3.86 x10-06	*LZTS1*	leucine_zipper_putative_tumor_suppressor_1
2.1157	4.53 x10-06	*ZDHHC11*	zinc_finger_DHHC-type_containing_11
3.6334	5.19 x10-06	*ADAM23*	ADAM_metallopeptidase_domain_23

Table S2A. Downregulated genes by LPS in PBMCs of PwD2 control *vs* non-diabetic control (N) (PwD2LPS/ NLPS).

S2B. Upregulated genes by LPS in PBMCs of PwD2 control *vs* non-diabetic control (N) (PwD2LPS/ NLPS).

(2A)

Log2Fold	P value	Gene Name	Gene Description
-9.5331	4.08 x10-42	*HLA-DQB1*	major_histocompatibility_complex_class_II_DQ_beta_1
-6.1463	7.43 x10-29	*HLA-DQA1*	major_histocompatibility_complex_class_II_DQ_alpha_1
-3.6818	1.19 x10-15	*CXCL10*	chemokine_(C-X-C_motif)_ligand_10
-3.6442	1.92 x10-14	*CXCL11*	chemokine_(C-X-C_motif)_ligand_11
-3.1043	4.07 x10-12	*LYZ*	lysozyme
-3.0349	2.11 x10-11	*HIST1H1C*	histone_cluster_1_H1c

-3.598	9.40 x10-11	***CCL8***	chemokine_(C-C_motif)_ligand_8
-3.1708	3.81 x10-10	***CXCL9***	chemokine_(C-X-C_motif)_ligand_9
-2.702	8.19 x10-10	***APOBEC3A***	apolipoprotein_B_mRNA_editing_enzyme_catalytic_polypeptide-3lik
-4.1992	4.27 x10-09	***AGAP7***	ArfGAP_with_GTPase_domain_ankyrin_repeat_and_PH_domain_7
-2.7381	4.52 x10-09	***SIGLEC1***	sialic_acid_binding_Ig-like_lectin_1_sialoadhesin
-2.6088	1.17 x10-08	***CCL4L2***	chemokine_(C-C_motif)_ligand_4-like_2
-2.6796	1.70 x10-08	***BCL2L14***	BCL2-like_14_(apoptosis_facilitator)
-2.7119	1.89 x10-08	***DACT1***	dapper_antagonist_of_beta-catenin_homolog_1_(Xenopus_laevis)
-3.6105	2.00 x10-08	***TMEM176B***	transmembrane_protein_176B
-3.0804	4.17 x10-08	***BATF2***	basic_leucine_zipper_transcription_factor_ATF-like_2
-2.5952	5.34 x10-08	***HSPA7***	heat_shock_70kDa_protein_7_(HSP70B)
-2.6021	7.78 x10-08	***MSR1***	macrophage_scavenger_receptor_1
-2.6733	8.90 x10-08	***NEXN***	nexilin_(F_actin_binding_protein)
-2.2636	1.50 x10-07	***OLR1***	oxidized_low_density_lipoprotein_(lectin-like)_receptor_1
-3.034	3.74 x10-07	***TMEM176A***	transmembrane_protein_176A
-2.2077	3.77 x10-07	***IFITM3***	interferon_induced_transmembrane_protein_3
2.4709	4.16 x10-07	***NLRP6***	NLR_family_pyrin_domain_containing_6
-5.0748	4.78 x10-07	***S100B***	S100_calcium_binding_protein_B
-3.5353	4.89 x10-07	***GAPDHP14***	glyceraldehyde-3-phosphate_dehydrogenase_pseudogene_14
-2.2171	5.66 x10-07	***CFL2***	cofilin_2_(muscle)
2.5471	6.89 x10-07	***CYB5R2***	cytochrome_b5_reductase_2
-2.1114	7.81 x10-07	***RSAD2***	radical_S-adenosyl_methionine_domain_containing_2
-2.6326	9.32 x10-07	***CLEC10A***	C-type_lectin_domain_family_10_member_A
-3.327	9.44 x10-07	***HIST1H1E***	histone_cluster_1_H1e
-4.9532	1.30 x10-06	***TBX3***	T-box_3

-2.7684	1.43 x10-06	***PLD4***	phospholipase_D_family_member_4
-2.1459	1.46 x10-06	***CISH***	cytokine_inducible_SH2-containing_protein
-7.7777	1.61 x10-06	***H2AFB1***	H2A_histone_family_member_B1
-2.0156	2.36 x10-06	***RPS26***	ribosomal_protein_S26
-2.0467	2.68 x10-06	***IFNG***	Interferon-gamma
-2.4592	2.74 x10-06	***ATP8B4***	ATPase_class_I_type_8B_member_4
-2.2452	2.74 x10-06	***FCGR2C***	Fc_fragment_of_IgG_low_affinity_IIc_receptor_for_(CD32)_(gene/pseu
-2.1233	2.85 x10-06	***TMEM156***	transmembrane_protein_156
-2.2295	3.33 x10-06	***XIRP1***	xin_actin-binding_repeat_containing_1
-2.3588	3.73 x10-06	***TRBV28***	T_cell_receptor_beta_variable_28
-2.4268	3.76 x10-06	***USP32P2***	ubiquitin_specific_peptidase_32_pseudogene_2
-2.3233	4.04 x10-06	***APCDD1L***	adenomatosis_polyposis_coli_down-regulated_1-like
-2.0685	4.52 x10-06	***FPR1***	formyl_peptide_receptor_1
-4.7733	5.25 x10-06	***PLGLB1***	plasminogen-like_B1
-2.4834	7.14 x10-06	***TMEM255A***	transmembrane_protein_255A
-2.4687	8.17 x10-06	***HS3ST3A1***	heparan_sulfate_(glucosamine)_3-O-sulfotransferase_3A1
-7.5475	8.28 x10-06	***SLC44A5***	solute_carrier_family_44_member_5

(2B)

**log2 Fold**	**P value**	**Gene Name**	**Gene Description**
6.4054	3.78E-26	***CCL3L3***	chemokine_(C-C motif)_ligand_3-like_3
4.7988	1.55E-22	***CCL4L1***	chemokine_(C-C motif)_ligand_4-like_1
5.305	2.55E-16	***FAM21B***	family_with_sequence_similarity_21_member_B
3.5521	1.08E-13	***MDGA1***	MAM_domain_containing_glycosylphosphatidylinositol_anchor_1
3.5359	7.98E-12	***AKR1C2***	aldo-keto_reductase_family_1_member_C2
5.3428	1.40E-11	***TRGV5P***	T_cell_receptor_gamma_variable_5P_(pseudogene)

3.03	6.88E-11	***DDX11***	DEAD/H_(Asp-Glu-Ala-Asp/His)_box_helicase_11
3.7414	1.02E-10	***RGPD2***	RANBP2-like_and_GRIP_domain_containing_2
4.4769	2.74E-09	***DDX11L10***	DEAD/H_(Asp-Glu-Ala-Asp/His)_box_helicase_11_like_10
3.1641	4.02E-08	***HLA-K***	Major histocompatibility_complex_class_I_K_(pseudogene)
8.1984	4.11E-08	***GSTM1***	glutathione_S-transferase_mu_1
2.6027	6.29E-08	***CNIH3***	cornichon_homolog_3_(Drosophila)
3.1379	9.18E-08	***NUAK1***	NUAK_family_SNF1-like_kinase_1
5.1963	9.80E-08	***ZNF683***	zinc_finger_protein_683
2.797	1.17E-07	***EVC***	Ellis_van_Creveld_syndrome
5.1616	1.33E-07	***PDZK1IP1***	PDZK1_interacting_protein_1
5.0897	2.51E-07	***LONRF2***	LON_peptidase_N-terminal_domain_and_ring_finger_2
2.5413	2.78E-07	***FMN1***	formin_1
7.8969	5.06E-07	***TDRD12***	tudor_domain_containing_12
2.2977	3.03E-07	***AKR1C1***	aldo-keto_reductase_family_1_member_C1
3.138	7.04E-07	***KRT1***	keratin_1
3.5999	9.87E-07	***IL5RA***	interleukin_5_receptor_alpha
2.1013	1.19E-06	***CD9***	CD9_molecule
7.7456	1.61E-06	***NME1***	NME/NM23_nucleoside_diphosphate_kinase_1
2.086	2.66E-06	***ZDHHC11B***	zinc_finger_DHHC-type_containing_11B
3.6511	5.19E-06	***COL5A2***	collagen_type_V_alpha_2
3.9741	7.63E-06	***MANEAL***	mannosidase_endo-alpha-like
2.8034	7.77E-06	***BEGAIN***	brain-enriched_guanylate_kinase-associated
2.3675	3.75E-06	***RGMA***	RGM_domain_family_member_A
2.3135	4.50E-06	***LZTS1***	leucine_zipper_putative_tumor_suppressor_1
2.0232	5.04E-06	***NRP1***	neuropilin_1

TABLE S3A. Downregulated genes by RES alone in PBMCs of PwD2 (PwD2RES/ PwD2C).

S3B. Upregulated genes by RES alone in PBMCs of PwD2 (PwD2RES/ PwD2C).
(3A)

Log2 Fold	p value	Gene Name	Gene Description
-3.6161	6.20×10^{-15}	***CCL2***	chemokine_(C-C_motif)_ligand_2
-3.4079	6.59×10^{-14}	***THBD***	thrombomodulin
-3.3311	1.89×10^{-13}	***CCL7***	chemokine_(C-C_motif)_ligand_7
-3.3307	3.63×10^{-12}	***CYP1B1-AS1***	CYP1B1_antisense_RNA_1
-2.9896	9.95×10^{-11}	***SPRY2***	sprouty_homolog_2_(Drosophila)
-2.7007	6.30×10^{-10}	***THBS1***	thrombospondin_1
-2.5997	2.34×10^{-09}	***CYP1B1***	cytochrome_P450_family_1_subfamily_B_polypeptide_1
-2.5361	5.56×10^{-09}	***FUCA1***	fucosidase_alpha-L-_1_tissue
-3.1882	7.76×10^{-09}	***CD180***	CD180_molecule
-2.5250	9.99×10^{-09}	***EGR2***	early_growth_response_2
-2.5006	1.75×10^{-08}	***RGL1***	ral_guanine_nucleotide_dissociation_stimulator-like_1
-3.8865	1.97×10^{-08}	***WNT5A***	wingless-type_MMTV_integration_site_family_member_5A
-2.4769	1.62×10^{-07}	***CYP1A1***	cytochrome_P450_family_1_subfamily_A_polypeptide_1
-2.7496	2.03×10^{-07}	***CNR2***	cannabinoid_receptor_2_(macrophage)
-2.2386	3.07×10^{-07}	***GPR68***	G_protein-coupled_receptor_68
-5.0330	6.64×10^{-07}	***CHRNA6***	cholinergic_receptor_nicotinic_alpha_6_(neuronal)
-2.2548	1.04×10^{-06}	***GAPT***	GRB2-binding_adaptor_protein_transmembrane
-2.2632	1.13×10^{-06}	***PTGFRN***	prostaglandin_F2_receptor_inhibitor
-2.1618	1.14×10^{-06}	***HIC1***	hypermethylated_in_cancer_1
-2.1423	1.20×10^{-06}	***RASAL1***	RAS_protein_activator_like_1_(GAP1_like)
-2.6084	1.64×10^{-06}	***FPR2***	formyl_peptide_receptor_2
-2.4933	2.59×10^{06}	***IL10***	interleukin_10
-2.0005	2.69×10^{-06}	***FCAR***	Fc_fragment_of_IgA_receptor_for
-2.0345	3.34×10^{-06}	***AHRR***	aryl-hydrocarbon_receptor_repressor

(3B)

Log2 Fold	p value	Gene Name	Gene Description
6.6045	5.78 x10^{-19}	***ITGAD***	integrin_alpha_D
3.0399	6.94 x10^{-12}	***NEAT1***	nuclear_paraspeckle_assembly_transcript_1_(non-protein_coding)
3.6886	1.79 x10^{-09}	***CRYM***	crystallin_mu
2.4904	4.47 x10^{-08}	***TMEM119***	transmembrane_protein_119
3.4724	4.94 x10^{-08}	***POPDC2***	popeye_domain_containing_2
2.5016	4.00 x10^{-07}	***C11orf96***	chromosome_11_open_reading_frame_96
3.0623	5.61 x10^{-07}	***COLQ***	Collagen like_tail_subunit_(single_strand_of_homotrimer) _of_asymmetri
3.0442	6.71 x10^{-07}	***MT1E***	metallothionein_1E
2.3501	1.42 x10^{-06}	***MS4A14***	membrane-spanning_4-domains_subfamily_A_member_14
2.0814	1.82 x10^{-06}	***KRCC1***	lysine-rich_coiled-coil_1
7.7259	2.40 x10^{-06}	***TAS1R1***	taste_receptor_type_1_member_1
3.6817	3.89 x10^{-06}	**PMEL**	premelanosome_protein

TABLE S4A. LPS-induced genes downregulated by RES in PBMCs from PwD2 (PwD2LPS + RES/PwD2 LPS).

S4B. LPS-induced genes upregulated by RES in PBMCs from PwD2 (PwD2LPS + RES/PwD2 LPS).

(4A)

log2 Fold	p value	Gene Name	Gene Description
-4.4053	1.37 x10^{-20}	***CYP1B1***	cytochrome_P450_family_1_subfamily_B_polypeptide_1
-4.4001	3.69 x10^{-18}	***CCL2***	chemokine_(C-C_motif)_ligand_2
-3.9942	8.94 x10^{-14}	***CYP1B1-AS1***	CYP1B1_antisense_RNA_1
-4.8564	1.28 x10^{-12}	***IL19***	interleukin_19
-3.7227	2.57 x10^{-12}	***GGT5***	gamma-glutamyltransferase_5
-3.1358	2.72 x10^{-12}	***CMPK2***	cytidine_monophosphate_(UMP-CMP)_kinase_2_mitochondrial
-3.0323	2.75 x10^{-11}	***CCL7***	chemokine_(C-C_motif)_ligand_7
-2.9855	3.00 x10^{-11}	***NEU4***	sialidase_4

-3.0878	4.21 x10^{-11}	***CSF2***	colony_stimulating_factor_2_(granulocyte-macrophage)
-2.9706	8.01 x10^{-11}	***GPR68***	G_protein-coupled_receptor_68
-2.8758	2.39 x10^{-10}	***EGR2***	early_growth_response_2
-3.1356	2.90 x10^{-10}	***THBD***	thrombomodulin
-2.7784	3.10 x10^{-10}	***OAS3***	2'-5'-oligoadenylate_synthetase_3_100kDa
-3.151	1.35 x10^{-09}	***ASB2***	ankyrin_repeat_and_SOCS_box_containing_2
-2.6445	2.31 x10^{-09}	***OAS1***	2'-5'-oligoadenylate_synthetase_1_40/46kDa
-3.3362	4.71 x10^{-09}	***PNPLA1***	patatin-like_phospholipase_domain_containing_1
-2.5477	4.92 x10^{-09}	***RSAD2***	radical_S-adenosyl_methionine_domain_containing_2
-8.466	5.75 x10^{-09}	***METTL21B***	methyltransferase_like_21B
-2.5733	7.40 x10^{-09}	***LAMB3***	laminin_beta_3
-2.806	1.16 x10^{-08}	***DDIT4L***	DNA-damage-inducible_transcript_4-like
-4.8395	1.37 x10^{-08}	***LAMA2***	laminin_alpha_2
-2.7705	1.75 x10^{-08}	***GAPT***	GRB2-binding_adaptor_protein_transmembrane
-2.5559	2.02 x10^{-08}	***IL36G***	interleukin_36_gamma
-3.8217	2.32 x10^{-08}	***LINC00677***	long_intergenic_non-protein_coding_RNA_677
-2.4186	3.51 x10^{-08}	***CD93***	CD93_molecule
-2.3682	5.22 x10^{-08}	***SAMD9L***	sterile_alpha_motif_domain_containing_9-like
-2.5256	7.12 x10^{-08}	***HIC1***	hypermethylated_in_cancer_1
-3.0828	7.39 x10^{-08}	***AC099552.4***	Uncharacterized_protein_
-2.3103	8.16 x10^{-08}	***HELZ2***	helicase_with_zinc_finger_2_transcriptional_coactivator
-2.3480	1.41 x10^{-07}	***USP18***	ubiquitin_specific_peptidase_18
-2.2817	1.44 x10^{-07}	***IFI6***	interferon_alpha-inducible_protein_6
-2.4184	1.64 x10^{-07}	***MGAM***	maltase-glucoamylase_(alpha-glucosidase)
-2.2383	1.93 x10^{-07}	***MX1***	myxovirus_(influenzavirus)_resistance_1_interferon-inducible_protei

-2.3391	2.53 x10^{-07}	***CSF1***	colony_stimulating_factor_1_(macrophage)
-2.6078	4.26 x10^{-07}	***SLC1A2***	solute_carrier_family_1_(glial_high_affinity_glutamate_transporter)
-2.1892	4.88 x10^{-07}	***AK4***	adenylate_kinase_4
-2.9314	4.93 x10^{-07}	***CNTLN***	centlein_centrosomal_protein
-2.1553	4.98 x10^{-07}	***SLC39A8***	solute_carrier_family_39_(zinc_transporter)_member_8
-2.9604	6.06 x10^{-07}	***CXCL11***	chemokine_(C-X-C_motif)_ligand_11
-2.2311	6.28 x10^{-07}	***AHRR***	aryl-hydrocarbon_receptor_repressor
-2.1349	6.50 x10^{-07}	***INHBA***	inhibin_beta_A
-2.4542	9.29 x10^{-07}	***FJX1***	four_jointed_box_1_(Drosophila)
-3.9841	9.57 x10^{-07}	***ABCA13***	ATP-binding_cassette_sub-family_A_(ABC1) member_13
-3.4302	9.87 x10^{-07}	***CAMK1G***	calcium/calmodulin-dependent_protein_kinase_IG
-7.8113	1.09 x10^{-06}	***RNF126P1***	ring_finger_protein_126_pseudogene_1
-2.7461	1.31 x10^{-06}	***CTD-2547L24.3***	HCG1816139; Uncharacterized_protein_
-2.4963	1.48 x10^{-06}	***IL10***	interleukin_10
-2.4762	1.67 x10^{-06}	***HMGA2***	high_mobility_group_AT-hook_2
-4.9803	1.83 x10^{-06}	***CXCL6***	chemokine_(C-X-C_motif)_ligand_6
-2.2489	2.17 x10^{-06}	***SYNPO2***	synaptopodin_2
-2.1337	2.27 x10^{-06}	***CXCL10***	chemokine_(C-X-C_motif)_ligand_10
-2.1185	2.38 x10^{-06}	***PDGFB***	platelet-derived_growth_factor_beta_polypeptide
-2.7226	2.94 x10^{-06}	***PRKAG2-AS1***	PRKAG2_antisense_RNA_1
-2.0032	3.11 x10^{-06}	***EPSTI1***	epithelial_stromal_interaction_1_(breast)
-2.5304	3.19 x10^{-06}	***GPRC5C***	G_protein-coupled_receptor_family_C_group_5_member_C
-2.0149	3.49 x10^{-06}	***ARHGEF11***	Rho_guanine_nucleotide_exchange_factor_(GEF)_11
-1.9805	3.50 x10^{-06}	***TPRA1***	transmembrane_protein_adipocyte_asscociated_1
-2.5153	3.66 x10^{-06}	***MACC1***	metastasis_associated_in_colon_cancer_1

-1.9918	4.17 x10^{-06}	***SLC16A6***	solute_carrier_family_16_member_6_(monocarboxylic_acid_transport
-2.0160	4.40 x10^{-06}	***DFNA5***	deafness_autosomal_dominant_5
-2.2061	4.42 x10^{-06}	***ETV7***	ets_variant_7
-2.0164	4.62 x10^{-06}	***DAGLA***	diacylglycerol_lipase_alpha
-1.9466	4.73 x10^{-06}	***SLC7A11***	solute_carrier_family_7_(anionic_amino_acid_transporter_light_chain
-2.0589	4.96 x10^{-06}	***GJA3***	gap_junction_protein_alpha_3_46kDa
-2.6497	5.55 x10^{-06}	***IL1RL2***	interleukin_1_receptor-like_2
-2.4418	6.25 x10^{-06}	***BLACE***	B-cell_acute_lymphoblastic_leukemia_expressed
-2.4374	7.26 x10^{-06}	***CACNA1A***	calcium_channel_voltage-dependent_P/Q_type_alpha_1A_subunit
-2.8209	7.68 x10^{-06}	***LRG1***	leucine-rich_alpha-2-glycoprotein_1
-2.5696	8.85 x10^{-06}	***XCR1***	chemokine_(C_motif)_receptor_1
-2.0097	9.63 x10^{-06}	***PARP16***	poly_(ADP-ribose)_polymerase_family_member_16
-1.8824	9.77 x10^{-06}	***XAF1***	XIAP_associated_factor_1
-1.9645	1.03 x10^{-05}	***RGL1***	ral_guanine_nucleotide_dissociation_stimulator-like_1
-2.5510	1.04 x10^{-05}	***IL36RN***	interleukin_36_receptor_antagonist
-1.8848	1.04 x10^{-05}	***HK2***	hexokinase_2
-1.9493	1.09 x10^{-05}	***RAB20***	RAB20_member_RAS_oncogene_family
-1.8656	1.10 x10^{-05}	***OAS2***	2'-5'-oligoadenylate_synthetase_2_69/71kDa
-2.3297	1.11 x10^{-05}	***TRIM16L***	tripartite_motif_containing_16-like
-2.3108	1.21 x10^{-05}	***OLIG1***	oligodendrocyte_transcription_factor_1
-7.5047	1.27 x10^{-05}	***CDH15***	cadherin_15_type_1_M-cadherin_(myotubule)
-2.0387	1.31 x10^{-05}	***PMP22***	peripheral_myelin_protein_22
-1.8436	1.46 x10^{-05}	***IFIT1***	interferon-induced_protein_with_tetratricopeptide_repeats_1

(4B)

**Log2 Fold**	**p value**	**Gene Name**	**Gene Description**
3.0386	1.63 x10^{-10}	***TMEM119***	transmembrane_protein_119

5.4503	4.11 x10^{-09}	***ZNF300P1***	zinc_finger_protein_300_pseudogene_1
2.642	1.07 x10^{-08}	***HPN***	hepsin
2.607	6.29 x10^{-08}	***MERTK***	c-mer_proto-oncogene_tyrosine_kinase
4.9299	4.78 x10^{-07}	***BTBD18***	BTB_(POZ)_domain_containing_18
2.2969	5.89 x10^{-07}	***DNAJC3-AS1***	DNAJC3_antisense_RNA_1_(head_to_head)
2.0618	2.65 x10^{-06}	***IL18***	interleukin_18_(interferon-gamma-inducing_factor)
1.9798	3.25 x10^{-06}	***NEAT1***	nuclear_paraspeckle_assembly_transcript_1_(non-protein_coding)
2.0199	3.38 x10^{-06}	***CPM***	carboxypeptidase_M
2.5202	8.68 x10^{-06}	***C9orf131***	chromosome_9_open_reading_frame_131
3.8515	1.06 x10^{-05}	***HNRNPA3P2***	heterogeneous_nuclear_ribonucleoprotein_A3_pseudogene_2
1.8804	1.09 x10^{-05}	***PSMD5***	proteasome_(prosome_macropain)_26S_subunit_non-ATPase_5
3.7669	2.06 x10^{-05}	***ELFN2***	extracellular_leucine-rich_repeat_and_fibronectin_type_III_domain_
2.0009	2.13 x10^{-05}	***RASL11A***	RAS-like_family_11_member_A
4.4016	3.37 x10^{-05}	***CYP46A1***	cytochrome_P450_family_46_subfamily_A_polypeptide_1
2.6633	4.81 x10^{-05}	***LINC00663***	long_intergenic_non-protein_coding_RNA_663
1.8835	6.07 x10^{-05}	***KCNC3***	potassium_voltage-gated_channel_Shaw-related_subfamily_3
2.0234	6.39 x10^{-05}	***TMEM52B***	transmembrane_protein_52B
4.1077	0.00016	***SLC13A4***	solute_carrier_family_13_(sodium/sulfate_symporters)_member_4
2.8054	0.00017	***LDHAL6A***	lactate_dehydrogenase_A-like_6A

TABLE S5A: Upregulated genes by LEC50 alone in PBMCs of PwD2 control (PwD2LEC50/ PwD2C).

S5B: Downregulated genes by LEC50 alone in PBMCs of PwD2 control (PwD2LEC50/ PwD2C).

(5A)

**log2 Fold**	**pValue**	**Gene Name**	**Gene Description**
10.307	9.53 x10^{-61}	***IL6***	interleukin_6_(interferon_beta_2)
10.906	8.58 x10^{-49}	***CSF3***	colony_stimulating_factor_3_(granulocyte)
9.0459	1.43 x10^{-48}	***CCL3L1***	chemokine_(C-C_motif)_ligand_3-like_1
8.0958	5.25 x10^{-46}	***IL1A***	interleukin_1_alpha
9.4089	5.87 x10^{-45}	***CCL3***	chemokine_(C-C_motif)_ligand_3
7.8232	3.46 x10^{-44}	***F3***	coagulation_factor_III_(thromboplastin_tissue_factor)
8.2237	1.02 x10^{-43}	***CCL4L1***	chemokine_(C-C_motif)_ligand_4-like_1

7.3835	7.18 x10-42	***CCL4***	chemokine_(C-C_motif)_ligand_4
7.7068	2.00 x10-41	***CCL20***	chemokine_(C-C_motif)_ligand_20
6.9967	3.33 x10-39	***PTGS2***	prostaglandin-endoperoxide_synthase_2_(prostaglandin_G/H_syn
7.1005	2.29 x10-37	***IRG1***	immunoresponsive_1_homolog_(mouse)
6.6831	4.04 x10-37	***IL1B***	interleukin_1_beta
6.7709	2.18 x10-36	***INHBA***	inhibin_beta_A
8.1400	1.29 x10-35	***CSF2***	colony_stimulating_factor_2_(granulocyte-macrophage)
7.4608	3.41 x10-33	***CCL3L3***	chemokine_(C-C_motif)_ligand_3-like_3
8.1741	1.15 x10-31	***IL36G***	interleukin_36_gamma
5.7800	5.96 x10-30	***TNFAIP6***	tumor_necrosis_factor_alpha-induced_protein_6
6.1046	6.25 x10-29	***ADORA2A-AS1***	ADORA2A_antisense_RNA_1
6.1539	1.20 x10-28	***DNAAF1***	dynein_axonemal_assembly_factor_1
5.8319	1.11 x10-27	***KCNJ2***	potassium_inwardly-rectifying_channel_subfamily_J_member_2
8.5007	3.51 x10-26	***ZNF259P1***	zinc_finger_protein_259_pseudogene_1
11.429	9.39 x10-26	***FRMD7***	FERM_domain_containing_7
11.315	5.30 x10-25	***RHCG***	Rh_family_C_glycoprotein
6.2422	3.63 x10-23	***KCNJ2-AS1***	KCNJ2_antisense_RNA_1_(head_to_head)
4.6248	4.75 x10-22	***TNF***	tumor_necrosis_factor
6.7537	8.13 x10-22	***INHBA-AS1***	INHBA_antisense_RNA_1
4.7181	1.47 x10-21	***SCN1B***	sodium_channel_voltage-gated_type_I_beta_subunit
5.3417	1.32 x10-20	***SYNPO2***	synaptopodin_2
5.1167	4.11 x10-19	***E2F7***	E2F_transcription_factor_7
5.6052	4.41 x10-18	***CCDC147-AS1***	CCDC147_antisense_RNA_1_(head_to_head)
6.532	6.49 x10-18	***HPN***	hepsin
3.908	2.28 x10-17	***CXCL2***	chemokine_(C-X-C_motif)_ligand_2

5.4819	2.47×10^{-17}	***CCL4L2***	chemokine_(C-C_motif)_ligand_4-like_2
4.2451	3.56×10^{-17}	***TNIP3***	TNFAIP3_interacting_protein_3
4.0761	9.52×10^{-17}	***PDSS1***	prenyl_(decaprenyl)_diphosphate_synthase_subunit_1
5.217	1.78×10^{-16}	***XCR1***	chemokine_(C_motif)_receptor_1
4.0903	1.98×10^{-16}	***PLD1***	phospholipase_D1_phosphatidylcholine-specific
6.945	2.99×10^{-16}	***EHF***	ets_homologous_factor
4.8889	3.93×10^{-16}	***CES1P1***	carboxylesterase_1_pseudogene_1
4.0178	5.76×10^{-16}	***IL7***	interleukin_7
3.8168	6.10×10^{-16}	***hsa-mir-146a***	hsa-mir-146a
3.6912	8.74×10^{-16}	***RIN2***	Ras_and_Rab_interactor_2
4.9908	9.54×10^{-16}	***C1QTNF1***	C1q_and_tumor_necrosis_factor_related_protein_1
4.8294	9.70×10^{-16}	***FLT1***	fms-related_tyrosine_kinase_1
3.6537	1.10×10^{-15}	***CXCL1***	Chemokine-(CX-C_motif)_ligand_1_(melanoma_growth_stimulati
4.5644	1.29×10^{-15}	***SLC22A1***	solute_carrier_family_22_(organic_cation_transporter)_member_1
3.5169	1.02×10^{-14}	***BCL2A1***	BCL2-related_protein_A1
3.5715	4.36×10^{-14}	***UPB1***	ureidopropionase_beta
4.7994	5.55×10^{-14}	***AL135998.1***	Uncharacterized_protein_
3.5031	7.16×10^{-14}	***ADORA2A***	adenosine_A2a_receptor
3.3452	9.35×10^{-14}	***IL8***	interleukin_8
5.4297	1.19×10^{-13}	***LINC00346***	long_intergenic_non-protein_coding_RNA_346
3.2946	1.97×10^{-13}	***CXCL3***	chemokine_(C-X-C_motif)_ligand_3
3.3606	2.08×10^{-13}	***SGPP2***	sphingosine-1-phosphate_phosphatase_2
3.3359	2.11×10^{-13}	***MIR155HG***	MIR155_host_gene_(non-protein_coding)
3.6356	2.47×10^{-13}	***ADRA2B***	adrenoceptor_alpha_2B
3.402	3.65×10^{-13}	***DFNA5***	deafness_autosomal_dominant_5

5.3396	3.77 x10-13	***LINC00515***	long_intergenic_non-protein_coding_RNA_515
3.2506	3.84 x10-13	***NFKBIZ***	nuclear_factor_of_kappa_light_polypeptide_gene_enhancer_in_B-c
3.2549	5.02 x10-13	***GJB2***	gap_junction_protein_beta_2_26kDa
3.211	8.85 x10-13	***RNF144B***	ring_finger_protein_144B
3.2489	1.29 x10-12	***SFR1***	SWI5-dependent_recombination_repair_1
3.1874	1.46 x10-12	***XAF1***	XIAP_associated_factor_1
3.3184	1.85 x10-12	***TEX14***	testis_expressed_14
4.5203	1.89 x10-12	***TSLP***	thymic_stromal_lymphopoietin
9.2001	3.82 x10-12	***PCNPP3***	PEST_containing_nuclear_protein_pseudogene_3
4.7195	4.08 x10-12	***IL21-AS1***	IL21_antisense_RNA_1
3.1193	4.29 x10-12	***CMPK2***	cytidine_monophosphate_(UMP-CMP)_kinase_2_mitochondrial
9.1767	4.84 x10-12	***IL12B***	interleukin_12B_(natural_killer_cell_stimulatory_factor_2_cytotoxic
9.1767	4.84 x10-12	***SERPINB7***	serpin_peptidase_inhibitor_clade_B_(ovalbumin)_member_7
3.1093	5.19 x10-12	***ADCY9***	adenylate_cyclase_9
3.061	5.54 x10-12	***NLRP3***	NLR_family_pyrin_domain_containing_3
3.4564	6.34 x10-12	***C11orf96***	chromosome_11_open_reading_frame_96
3.9058	9.28 x10-12	***GCKR***	glucokinase_(hexokinase_4) regulator
3.0075	1.35 x10-11	***ISG15***	ISG15_ubiquitin-like_modifier
3.1755	1.53 x10-11	***ELOVL7***	ELOVL_fatty_acid_elongase_7
3.0172	1.68 x10-11	***IFIT1***	interferon-induced_protein_with_tetratricopeptide_repeats_1
3.2052	3.01 x10-11	***MGAM***	maltase-glucoamylase_(alpha-glucosidase)
2.9649	3.03 x10-11	***ITGB8***	integrin_beta_8
3.5072	3.75 x10-11	***C1orf61***	chromosome_1_open_reading_frame_61
2.9801	4.36 x10-11	***TRIP10***	thyroid_hormone_receptor_interactor_10
2.9333	5.45 x10-11	***MFSD2A***	major_facilitator_superfamily_domain_containing_2A

4.1243	7.24 x10-11	***ABCA6***	ATP-binding_cassette_sub-family_A_(ABC1)_member_6
3.9607	8.80 x10-11	***GBP1P1***	guanylate_binding_protein_1_interferon-inducible_pseudogene_1
4.8672	1.06 x10-10	***BLACE***	B-cell_acute_lymphoblastic_leukemia_expressed
8.8612	1.30 x10-10	***ADAD2***	adenosine_deaminase_domain_containing_2
2.8166	1.56 x10-10	***SLC12A7***	solute_carrier_family_12_(potassium/chloride_transporters)_memb
3.9041	1.83 x10-10	***OSR2***	odd-skipped_related_2_(Drosophila)
2.7918	2.05 x10-10	***GRAMD1A***	GRAM_domain_containing_1A
2.7847	2.13 x10-10	***EREG***	epiregulin
2.7856	2.14 x10-10	***ACSL1***	acyl-CoA_synthetase_long-chain_family_member_1
2.8059	2.32 x10-10	***KANK1***	KN_motif_and_ankyrin_repeat_domains_1
2.9368	2.58 x10-10	***GPX3***	glutathione_peroxidase_3_(plasma)
2.7394	3.94 x10-10	***PELI1***	pellino_E3_ubiquitin_protein_ligase_1
8.7387	5.21 x10-10	***CFB***	complement_factor_B
3.4864	5.54 x10-10	***PNPLA1***	patatin-like_phospholipase_domain_containing_1
2.7102	5.59 x10-10	***SERPINB2***	serpin_peptidase_inhibitor_clade_B_(ovalbumin)_member_2
2.7238	5.83 x10-10	***DRAM1***	DNA-damage_regulated_autophagy_modulator_1
5.7199	6.95 x10-10	***IDO2***	indoleamine_23-dioxygenase_2
2.6997	7.15 x10-10	***MX1***	myxovirus_(influenza virus)_resistance_1_interferon-inducible_pr
2.6934	7.33 x10-10	***IFIT3***	interferon-induced_protein_with_tetratricopeptide_repeats_3
3.4286	7.76 x10-10	***HMGA2***	high_mobility_group_AT-hook_2
2.8585	1.12 x10-09	***KIAA1199***	KIAA1199
2.6486	1.26 x10-09	***TRAF1***	TNF_receptor-associated_factor_1
2.8879	1.26 x10-09	***LAMP3***	lysosomal associated_membrane_protein_3
3.1962	1.53 x10-09	***LINC00854***	long_intergenic_non-protein_coding_RNA_854
2.8002	1.65 x10-09	***USP18***	ubiquitin_specific_peptidase_18

3.3902	1.67 x10-09	***FJX1***	four_jointed_box_1_(Drosophila)
8.6047	1.68 x10-09	***PLCB4***	phospholipase_C_beta_4
2.6392	2.06 x10-09	***IFIT5***	interferon-induced_protein_with_tetratricopeptide_repeats_5
2.6708	2.16 x10-09	***OAS1***	2'-5'-oligoadenylate_synthetase_1_40/46kDa
2.8157	2.36 x10-09	***FFAR2***	free_fatty_acid_receptor_2
2.6017	2.52 x10-09	***CCRL2***	chemokine_(C-C motif)_receptor-like_2
3.3724	2.62 x10-09	***FGF13***	fibroblast_growth_factor_13
3.8067	2.82 x10-09	***FNDC4***	fibronectin_type_III_domain_containing_4
2.609	2.98 x10-09	***PILRA***	paired_immunoglobin-like_type_2_receptor_alpha
2.5773	3.36 x10-09	***GCH1***	GTP_cyclohydrolase_1
2.5669	6.47 x10-09	***PSTPIP2***	proline-serine-threonine_phosphatase_interacting_protein_2
2.7305	6.75 x10-09	***WNT5A***	wingless-type_MMTV_integration_site_family_member_5A
4.0683	7.95 x10-09	***OR2B11***	olfactory_receptor_family_2_subfamily_B_member_11
2.5049	9.95 x10-09	***NCR3LG1***	natural_killer_cell_cytotoxicity_receptor_3_ligand_1
8.4176	1.09 x10-08	***LINC00158***	long_intergenic_non-protein_coding_RNA_158
5.4312	1.09 x10-08	***NRBF2P2***	nuclear_receptor_binding_factor_2_pseudogene_2
2.8447	1.21 x10-08	***PLAU***	plasminogen_activator_urokinase
2.5415	1.24 x10-08	***IL2RA***	interleukin_2_receptor_alpha
2.4653	1.32 x10-08	***G0S2***	G0/G1switch_2
2.6543	1.35 x10^{08}	***ADTRP***	androgen-dependent_TFPI-regulating_protein
2.6389	1.35 x10^{08}	***IFI44L***	interferon-induced_protein_44-like
2.7018	1.63 x10-08	***CREB5***	cAMP_responsive_element_binding_protein_5
2.4375	1.75 x10-08	***IL1RN***	interleukin_1_receptor_antagonist
2.8995	1.80 x10-08	***CDK1***	cyclin-dependent_kinase_1
5.3490	2.10 x10-08	***GPIHBP1***	glycosylphosphatidylinositol_anchored_high_density_lipoprotein

8.3354	2.10 x10-08	***NBEAP1***	neurobeachin_pseudogene_1
2.8124	2.17 x10-08	***EDN1***	endothelin_1
2.3957	3.00 x10-08	***HELZ2***	helicase_with_zinc_finger_2_transcriptional_coactivator
2.4358	3.17 x10-08	***HSH2D***	hematopoietic_SH2_domain_containing
2.4014	3.75 x10-08	***HS3ST3B1***	heparan_sulfate_(glucosamine)_3-O-sulfotransferase_3B1
2.4546	3.87 x10-08	***IL15RA***	interleukin_15_receptor_alpha
2.5173	4.27 x10-08	***CD274***	CD274_molecule
2.4282	4.89 x10-08	***CKB***	creatine_kinase_brain
2.7863	5.17 x10-08	***ZP3***	zona_pellucida_glycoprotein_3_(sperm_receptor)
2.3257	6.55 x10-08	***SOD2***	superoxide_dismutase_2_mitochondrial
2.3291	6.87 x10-08	***OAS2***	2'-5'-oligoadenylate_synthetase_2_69/71kDa
4.6030	7.22 x10-08	***LAD1***	ladinin_1
8.2025	8.20 x10-08	***RANBP3L***	RAN_binding_protein_3-like
4.5646	9.80 x10-08	***NR5A2***	nuclear_receptor_subfamily_5_group_A_member_2
2.3007	1.14 x10-07	***OAS3***	2'-5'-oligoadenylate_synthetase_3_100kDa
2.2971	1.16 x10-07	***ARL5B***	ADP-ribosylation_factor-like_5B
5.1690	1.17 x10-07	***PRG4***	proteoglycan_4
3.3055	1.26 x10-07	***FANK1***	fibronectin_type_III_and_ankyrin_repeat_domains_1
2.3492	1.29 x10-07	***HDAC9***	histone_deacetylase_9
2.7236	1.30 x10-07	***FERMT2***	fermitin_family_member_2
2.4246	1.36 x10-07	***ALPK2***	alpha kinase_2
2.2721	1.67 x10-07	***DHX58***	DEXH_(Asp-Glu-X-His_box_polypeptide_58
8.1066	1.67 x10-07	***MMP10***	matrix_metallopeptidase_10_(stromelysin_2)
5.1203	1.67 x10-07	***TNFSF15***	tumor_necrosis_factor_(ligand)_superfamily_member_15
3.2198	1.91 x10-07	***BATF3***	basic_leucine_zipper_transcription_factor_ATF-like_3

2.7659	2.07 x10^{-07}	***RGMA***	RGM_domain_family_member_A
2.2289	2.13 x10^{-07}	***SLC2A6***	solute_carrier_family_2_(facilitated_glucose_transporter)_member
2.3043	2.35 x10^{-07}	***AKR1C1***	aldo-keto_reductase_family_1_member_C1
2.2894	2.62 x10^{-07}	***CRLF2***	cytokine_receptor-like_factor_2
2.2025	2.88 x10^{-07}	***PMAIP1***	phorbol-12-myristate-13-acetate-induced_protein_1
2.2064	2.99 x10^{-07}	***GBP1***	guanylate_binding_protein_1_interferon-inducible
2.9352	3.18 x10^{-07}	***STON2***	stonin_2
2.6211	3.43 x10^{-07}	***SLC1A2***	solute_carrier_family_1_(glial_high_affinity_glutamate_transporter
2.2172	3.54 x10^{-07}	***CLEC4E***	C-type_lectin_domain_family_4_member_E
2.1851	3.56 x10^{-07}	***SLAMF7***	SLAM_family_member_7
2.2343	3.65 x10^{-07}	***TMEM106A***	transmembrane_protein_106A
2.3337	4.49 x10^{-07}	***SMCO4***	single-pass_membrane_protein_with_coiled-coil_domains_4
2.8294	4.52 x10^{-07}	***LINC00677***	long_intergenic_non-protein_coding_RNA_677
4.0508	4.77 x10^{-07}	***TNRC18P1***	TNRC18P1
2.6667	4.99 x10^{-07}	***NEURL3***	neuralized_homolog_3_(Drosophila)_pseudogene
2.3537	7.95 x10^{-07}	***C12orf61***	chromosome_12_open_reading_frame_61
2.118	8.02 x10^{-07}	***IFI44***	interferon-induced_protein_44
2.3505	8.30 x10^{-07}	***GGT5***	gamma-glutamyltransferase_5
2.841	8.4 x10^{-07}	***CASP5***	caspase_5_apoptosis-related_cysteine_peptidase
2.1062	8.50 x10^{-07}	***SOCS3***	suppressor_of_cytokine_signaling_3
2.2455	1.02 x10^{06}	***RBKS***	ribokinase
4.907	1.09 x10^{-06}	***USP12-AS2***	USP12_antisense_RNA_2_(head_to_head)
2.6375	1.14 x10^{-06}	***DNAJC3-AS1***	DNAJC3_antisense_RNA_1_(head_to_head)
2.2065	1.17 x10^{-06}	***MRPS24***	mitochondrial_ribosomal_protein_S24
4.263	1.30 x10^{-06}	***WNT5B***	wingless-type_MMTV_integration_site_family_member_5B

2.2624	1.33×10^{-06}	***AKR1C2***	aldo-keto_reductase_family_1_member_C2
2.8036	1.52×10^{-06}	***IL36RN***	interleukin_36_receptor_antagonist
2.0558	1.58×10^{-06}	***MX2***	myxovirus_(influenza_virus) resistance_2_(mouse)
2.7074	1.84×10^{-06}	***COL17A1***	collagen_type_XVII_alpha_1
2.0552	1.86×10^{-06}	***SAMD9L***	sterile_alpha_motif_domain_containing_9-like
2.9696	1.86×10^{-06}	***WDR96***	WD_repeat_domain_96
2.539	2.19×10^{-06}	***MACC1***	metastasis_associated_in_colon_cancer_1
2.2073	2.34×10^{-06}	***HLA-L***	major_histocompatibility_complex_class_I_L_(pseudogene)
2.4227	2.50×10^{-06}	***F8***	coagulation_factor_VIII_procoagulant_component
2.0702	2.93×10^{-06}	***PDGFB***	platelet-derived_growth_factor_beta_polypeptide
2.8828	2.96×10^{-06}	***TULP2***	tubby_like_protein_2
2.0905	3.01×10^{-06}	***ASTL***	astacin-like_metallo-endopeptidase_(M12_family)
7.7098	3.61×10^{-06}	***BCL2L14***	BCL2-like_14_(apoptosis_facilitator)
7.7098	3.61×10^{-06}	***MTND1P5***	MT-ND1_pseudogene_5
1.9881	3.70×10^{-06}	***NEU4***	sialidase_4
2.4029	3.74×10^{-06}	***LEKR1***	leucine_glutamate_and_lysine_rich_1
2.1197	3.80×10^{-06}	***CDC42BPG***	CDC42_binding_protein_kinase_gamma_(DMPK-like)
2.2308	3.88×10^{-06}	***CHAC1***	ChaC_cation_transport_regulator_homolog_1_(E._coli)
1.9832	4.15×10^{-06}	***TBC1D9***	TBC1_domain_family_member_9_(with_GRAM_domain)
1.9951	4.30×10^{-06}	***ZNF697***	zinc_finger_protein_697
1.9579	4.35×10^{-06}	***CLIC4***	chloride_intracellular_channel_4
2.1423	4.68×10^{-06}	***IL18***	interleukin_18_(interferon-gamma-inducing_factor)
2.0418	4.72×10^{-06}	***ADORA2B***	adenosine_A2b_receptor
4.0573	5.25×10^{-06}	***MUC6***	mucin_6_oligomeric_mucus/gel-forming
2.0552	5.28×10^{-06}	***BRE***	brain_and_reproductive_organ-expressed_(TNFRSF1A_modulator)

4.6566	5.45 x10-06	***C9orf24***	chromosome_9_open_reading_frame_24
3.758	5.52 x10-06	***CCL18***	chemokine_(C-C_motif) _ligand_18_(pulmonary_and_activation-re
1.9256	6.16 x10-06	***EIF1B***	eukaryotic_translation_initiation_factor_1B
2.0152	6.25 x10-06	***APOL1***	apolipoprotein_L_1
2.2173	6.42 x10-06	***CLEC12A***	C-type_lectin_domain_family_12_member_A
1.9596	6.43 x10-06	***PHLDB1***	pleckstrin_homology-like_domain_family_B_member_1
2.2601	6.44 x10-06	***ZEB2-AS1***	ZEB2_antisense_RNA_1
1.9124	6.65 x10-06	***AQP9***	aquaporin_9
3.0108	6.75 x10-06	***ANKRD18DP***	ankyrin_repeat_domain_18D_pseudogene
2.0269	6.96 x10-06	***IRF9***	interferon_regulatory_factor_9
1.9181	7.38 x10-06	***OGFRL1***	opioid_growth_factor_receptor-like_1
2.32	7.54 x10-06	***CFLAR-AS1***	CFLAR_antisense_RNA_1
2.5439	7.57 x10-06	***CARD17***	caspase_recruitment_domain_family_member_17
2.3074	7.94 x10-06	***TBC1D30***	TBC1_domain_family_member_30
1.8951	8.36 x10-06	***PRDM8***	PR_domain_containing_8
1.9052	8.73 x10-06	***TNFSF8***	tumor_necrosis_factor_(ligand)_superfamily_member_8
1.883	9.37 x10-06	***ETS2***	v-ets_erythroblastosis_virus_E26_oncogene_homolog_2_(avian)
2.7575	9.47 x10-06	***4-Sep***	septin_4
1.8818	9.64 x10-06	***MAP3K8***	mitogen-activated_protein_kinase_kinase_kinase_8
1.8694	1.07 x10-05	***IFIT2***	interferon-induced_protein_with_tetratricopeptide_repeats_2
1.9904	1.08 x10-05	***SERPINE2***	serpin_peptidase_inhibitor_clade_E_(nexin_plasminogen_activato
1.865	1.08 x10-05	***TNFAIP2***	tumor_necrosis_factor_alpha-induced_protein_2
1.9617	1.09 x10-05	***ADAMDEC1***	ADAM-like_decysin_1
3.1581	1.12 x10-05	***CHRNB2***	cholinergic_receptor_nicotinic_beta_2_(neuronal)
2.9406	1.12 x10-05	***RNF223***	ring_finger_protein_223

1.8679	1.28 x10-05	***DDX58***	DEAD_(Asp-Glu-Ala-Asp)_box_polypeptide_58
1.8487	1.30 x10-05	***DUSP2***	dual_specificity_phosphatase_2
1.8393	1.41 x10-05	***PNRC1***	proline-rich_nuclear_receptor_coactivator_1
1.8452	1.44 x10-05	***TRIM22***	tripartite_motif_containing_22
1.8592	1.45 x10-05	***RIPK2***	receptor-interacting_serine-threonine_kinase_2
1.9499	1.50 x10-05	***DLGAP1-AS2***	DLGAP1_antisense_RNA_2
2.3502	1.53 x10-05	***CAMK1G***	calcium/calmodulin-dependent_protein_kinase_IG
1.8549	1.60 x10-05	***GADD45A***	growth_arrest_and_DNA-damage-inducible_alpha
1.8219	1.74 x10-05	***MAFF***	v-maf_musculoaponeurotic_fibrosarcoma_oncogene_homolog_F_
1.8161	1.91 x10-05	***RSAD2***	radical_S-adenosyl_methionine_domain_containing_2
1.804	2.01 x10-05	***IER3***	immediate_early_response_3
1.8603	2.04 x10-05	***CLCF1***	cardiotrophin-like_cytokine_factor_1
1.802	2.05 x10-05	***CD83***	CD83_molecule
1.7879	2.46 x10-05	***TJP2***	tight_junction_protein_2
1.8352	2.51 x10-05	***ASNS***	asparagine_synthetase_(glutamine-hydrolyzing)
1.78	2.76 x10-05	***P2RX4***	purinergic_receptor_P2X_ligand-gated_ion_channel_4
1.776	2.77 x10-05	***TXN***	thioredoxin
1.9432	2.80 x10-05	***MTRNR2L8***	MT-RNR2-like_8
7.4218	3.05 x10-05	***MYRIP***	myosin_VIIA_and_Rab_interacting_protein
2.6203	3.18 x10-05	***LINC00299***	long_intergenic_non-protein_coding_RNA_299
2.1665	3.20 x10-05	***COL24A1***	collagen_type_XXIV_alpha_1
3.1376	3.21 x10-05	***BMP2***	bone_morphogenetic_protein_2
1.7417	3.68 x10-05	***NFKBIA***	nuclear_factor_of_kappa_light_polypeptide_gene_enhancer_in_B-c
1.7408	3.73 x10-05	***SERPINB9***	serpin_peptidase_inhibitor_clade_B_(ovalbumin)_member_9
2.3242	3.74 x10-05	***RALGAPA1P***	Ral_GTPase_activating_protein_alpha_subunit_1_(catalytic)_pseu

2.3544	3.75 x10-05	***MUC1***	mucin_1_cell_surface_associated
2.0227	3.91 x10-05	***SLC24A4***	solute_carrier_family_24_(sodium/potassium/calcium_exchanger)
2.0221	4.00 x10-05	***HAS1***	hyaluronan_synthase_1
1.8143	4.04 x10-05	***ULK2***	unc-51-like_kinase_2_(C._elegans)
2.06	4.27 x10-05	***RFX8***	RFX_family_member_8_lacking_RFX_DNA_binding_domain
1.7658	4.28 x10-05	***NXT2***	nuclear_transport_factor_2-like_export_factor_2
1.7406	4.37 x10-05	***C1orf122***	chromosome_1_open_reading_frame_122
1.7268	4.72 x10-05	***MIAT***	myocardial_infarction_associated_transcript_(non-protein_coding)
2.6778	4.98 x10-05	***KLLN***	killin_p53-regulated_DNA_replication_inhibitor

(5B)

**Log2 Fold**	**p value**	**Gene Name**	**Gene Description**
-4.4217	1.76 x10-20	***HMOX1***	heme_oxygenase_(decycling)_1
-4.6815	3.84 x10-20	***DHRS9***	dehydrogenase/reductase_(SDR_family)_member_9
-4.2966	3.12 x10-18	***LRRC25***	leucine_rich_repeat_containing_25
-4.8123	1.53 x10-17	***SDS***	serine_dehydratase
-3.9053	1.86 x10-16	***C5AR2***	complement_component_5a_receptor_2
-3.7536	3.83 x10-16	***FUCA1***	fucosidase_alpha-L-_1_tissue
-4.3977	1.47 x10-14	***PTGFRN***	prostaglandin_F2_receptor_inhibitor
-3.4719	3.22 x10-14	***THBD***	thrombomodulin
-3.5253	1.39 x10-13	***RAI14***	retinoic_acid_induced_14
-3.5601	9.87 x10-13	***OLIG1***	oligodendrocyte_transcription_factor_1
-3.1795	1.74 x10-12	***CCR1***	chemokine_(C-C_motif)_receptor_1
-3.5173	1.59 x10-11	***IFI30***	interferon_gamma-inducible_protein_30
-2.9099	6.29 x10-11	***CEBPD***	CCAAT/enhancer_binding_protein_(C/EBP)_delta
-3.0057	1.69 x10-10	***CSF1R***	colony_stimulating_factor_1_receptor

-3.1064	1.82 x10-10	***CD163***	CD163_molecule
-2.786	2.24 x10-10	***C5AR1***	complement_component_5a_receptor_1
-3.1808	4.20 x10-10	***TMEM51***	transmembrane_protein_51
-2.798	4.61 x10-10	***CXXC5***	CXXC_finger_protein_5
-2.7977	5.21 x10-10	***TIMP2***	TIMP_metallopeptidase_inhibitor_2
-2.942	5.87 x10-10	***SERPINE1***	serpin_peptidase_inhibitor_clade_E_(nexin_plasminogen_activator_i
-4.5156	7.20 x10-10	***MYCL1***	vmyc_myelocytomatosis_viral_oncogene_homolog_1_lung_carcinom
-2.7515	7.96 x10-10	***FGL2***	fibrinogen-like_2
-2.8539	1.26 x10-09	***CYP27A1***	cytochrome_P450_family_27_subfamily_A_polypeptide_1
-2.6468	1.41 x10-09	***ZNF385A***	zinc_finger_protein_385A
-4.8689	1.44 x10-09	***GPR162***	G_protein-coupled_receptor_162
-3.1504	1.79 x10-09	***OLIG2***	oligodendrocyte_lineage_transcription_factor_2
-2.5889	3.58 x10-09	***ENG***	endoglin
-8.7259	4.11 x10-09	***LILRP1***	leukocyte_immunoglobulin-like_receptor_pseudogene_1
-2.5731	5.02 x10-09	***CD14***	CD14_molecule
-2.6413	5.27 x10-09	***SLC37A2***	solute_carrier_family_37_(glycerol-3-phosphate_transporter)_membe
-2.6119	9.70 x10-09	***CD300LB***	CD300_molecule-like_family_member_b
-3.1249	1.19 x10-08	***NOTCH3***	notch_3
-4.2559	1.21 x10-08	***PDK4***	pyruvate_dehydrogenase_kinase_isozyme_4
-2.5954	1.30 x10-08	***RASAL1***	RAS_protein_activator_like_1_(GAP1_like)
-2.4241	2.33 x10-08	***MPEG1***	macrophage_expressed_1
-3.1322	2.71 x10-08	***KANK2***	KN_motif_and_ankyrin_repeat_domains_2
-2.8967	2.89 x10-08	***CLDN5***	claudin_5
-2.5569	3.91 x10-08	***NTSR1***	neurotensin_receptor_1_(high_affinity)
-2.788	4.67 x10-08	***CD101***	CD101_molecule

-2.4158	2.76×10^{-07}	***NFAM1***	NFAT_activating_protein_with_ITAM_motif_1
-2.2696	3.14×10^{-07}	***PNKD***	paroxysmal_nonkinesigenic_dyskinesia
-2.1912	3.16×10^{-07}	***SEMA6B***	sema_domain_transmembrane_domain_(TM)_and_cytoplasmic_dom
-2.6656	4.69×10^{-07}	***ELFN1***	extracellular_leucine-rich_repeat_and_fibronectin_type_III_domain_c
-2.8085	5.13×10^{-07}	***CNR2***	cannabinoid_receptor_2_(macrophage)
-2.1609	6.41×10^{-07}	***FAM214B***	family_with_sequence_similarity_214_member_B
-2.5167	8.83×10^{-07}	***PRAM1***	PML-RARA_regulated_adaptor_molecule_1
-2.3891	9.81×10^{-07}	***KCNJ15***	potassium_inwardly-rectifying_channel_subfamily_J_member_15
-2.9637	9.92×10^{-07}	***EDNRB***	endothelin_receptor_type_B
-2.2355	1.17×10^{-06}	***ALDH3B1***	aldehyde_dehydrogenase_3_family_member_B1
-2.3579	1.23×10^{-06}	***DSC2***	desmocollin_2
-2.0691	1.66×10^{-06}	***PLIN3***	perilipin_3
-2.1553	1.89×10^{-06}	***RIN1***	Ras_and_Rab_interactor_1
-2.0222	2.34×10^{-06}	***TNS1***	tensin_1
-2.0165	2.71×10^{-06}	***TNFRSF1A***	tumor_necrosis_factor_receptor_superfamily_member_1A
-2.0736	2.77×10^{-06}	***SLC31A1***	solute_carrier_family_31_(copper_transporters)_member_1
-2.1265	2.95×10^{-06}	***INSR***	insulin_receptor
-2.1318	3.09×10^{-06}	***CDCP1***	CUB_domain_containing_protein_1
-2.2406	3.31×10^{-06}	***AVPI1***	arginine_vasopressin-induced_1
-2.3435	3.40×10^{-06}	***FPR3***	formyl_peptide_receptor_3
-2.0648	3.52×10^{-06}	***NCEH1***	neutral_cholesterol_ester_hydrolase_1
-1.9767	3.59×10^{-06}	***FAM129B***	family_with_sequence_similarity_129_member_B
-2.0984	3.65×10^{-06}	***GPNMB***	glycoprotein_(transmembrane)_nmb
-3.3473	4.11×10^{-06}	***RTN4RL2***	reticulon_4_receptor-like_2
-2.0073	4.21×10^{-06}	***TBC1D2***	TBC1_domain_family_member_2

-2.1625	4.64 x10-06	***PFKFB4***	6-phosphofructo-2-kinase/fructose-26-biphosphatase_4
-2.081	5.68 x10-06	***HBEGF***	heparin-binding_EGF-like_growth_factor
-1.9477	5.81 x10-06	***LYZ***	lysozyme
-2.0555	6.20 x10-06	***FHL3***	four_and_a_half_LIM_domains_3
-2.1669	6.23 x10-06	***EPHB2***	EPH_receptor_B2
-1.9255	7.31 x10-06	***MAPKAPK3***	mitogen-activated_protein_kinase-activated_protein_kinase_3
-1.992	7.95 x10-06	***SEMA3F***	sema_domain_immunoglobulin_domain_(Ig)_short_basic_domain_se
-1.9496	8.07 x10-06	***SLC17A5***	solute_carrier_family_17_(anion/sugar_transporter)_member_5
-1.9127	9.43 x10-06	***BMF***	Bcl2_modifying_factor
-1.9538	9.50 x10-06	***DAB2***	Dab_mitogen-responsive_phosphoprotein_homolog_2_(Drosophila)
-1.879	1.01 x10-05	***SLC16A3***	solute_carrier_family_16_member_3_(monocarboxylic_acid_transport
-1.8986	1.02 x10-05	***AGPAT9***	1-acylglycerol-3-phosphate_O-acyltransferase_9
-1.8825	1.02 x10-05	***ALAS1***	aminolevulinate_delta-_synthase_1
-1.8793	1.04 x10-05	***RAP2B***	RAP2B_member_of_RAS_oncogene_family
-1.9656	1.10 x10-05	***NDST1***	N-deacetylase/N-sulfotransferase_(heparan_glucosaminyl)_1
-1.9068	1.13 x10-05	***ACVR1***	activin_A_receptor_type_I
-1.9349	1.25 x10-05	***FCGR3A***	Fc_fragment_of_IgG_low_affinity_IIIa_receptor_(CD16a)
-2.060	1.37 x10-05	***CYP1A1***	cytochrome_P450_family_1_subfamily_A_polypeptide_1
-2.3274	1.48 x10-05	***CD180***	CD180_molecule
-1.8517	1.62 x10-05	***CCL2***	chemokine_(C-C_motif)_ligand_2
-2.4822	1.77 x10-05	***SDSL***	serine_dehydratase-like
-1.8725	1.78 x10-05	***ZBTB7B***	zinc_finger_and_BTB_domain_containing_7B
-2.3053	1.87 x10-05	***CABLES1***	Cdk5_and_Abl_enzyme_substrate_1
-2.1653	2.19 x10-05	***HTRA1***	HtrA_serine_peptidase_1
-1.7996	2.31 x10-05	***CCL7***	chemokine_(C-C_motif)_ligand_7

-1.8833	2.34 x10-05	***DTX4***	deltex_homolog_4_(Drosophila)
-2.3024	2.50 x10-05	***TRNP1***	TMF1-regulated_nuclear_protein_1
-1.8358	3.01 x10-05	***PHF23***	PHD_finger_protein_23
-1.8351	3.03 x10-05	***C10orf128***	chromosome_10_open_reading_frame_128
-7.3244	3.05 x10-05	***PRR11***	proline_rich_11
-1.7695	3.08 x10-05	***PLEKHO2***	pleckstrin_homology_domain_containing_family_O_member_2
-1.8403	3.35 x10-05	***FLVCR2***	feline_leukemia_virus_subgroup_C_cellular_receptor_family_membe
-1.7535	3.38 x10-05	***ZMIZ1***	zinc_finger_MIZ-type_containing_1
-1.7858	3.46 x10-05	***EEPD1***	endonuclease/exonuclease/phosphatase_family_domain_containing_1
-1.8039	3.68 x10-05	***RGS19***	regulator_of_G-protein_signaling_19
-1.7774	4.16 x10-05	***MBOAT7***	membrane_bound_O-acyltransferase_domain_containing_7
-1.7902	4.36 x10-05	***SERTAD3***	SERTA_domain_containing_3
-1.7313	4.51 x10-05	***MYO1F***	Myosin IF
-1.9028	4.97 x10-05	***LILRA2***	leukocyte_immunoglobulin-like_receptor_subfamily_A_(with_TM_

Table S6. Gene expression of transcription factors in A. PBMCs from PwD2 (D) and B. PBMCs from non-diabetics (N).

(6A) Transcription factor gene expression in PwD2*.

DC	DRES	DLPS	DLPS+RES	DLEC10	DLEC10+LPS	DLEC50	DLEC50+LPS
TBX3-	***EGR2-***	*HDAC9*	*NFKBIA*	*HDAC9*	*HDAC9*	*HDAC9*	*HDAC9*
NRG1-	***HIC1-***	***NOTCH3-***	*GADD45A*	*NOTCH3-*	*TRIM5*	*NOTCH3-*	*NOTCH3*
		VDR-	***EGR2-***	*EHF*	*EHF*	*NFKBIA*	*VDR-*
		TRIM5	***IRF5-***	*ETS2*	*PML*	*GADD45A*	*TRIM5*
		EHF	*EHF*	*E2F7*	*ETS2*	*EHF*	*EHF*
		PML	*E2F7*	*OLIG2-*	*E2F7*	*ETS2*	*PML*
		E2F7	***ZNF668-***		*IRF7*	*E2F7*	*ETS2*
		IRF7	***HIC1-***		*OLIG2-*	*MAFF*	*E2F7*
		OLIG2-	***OLIG2-***		*IRF9*	*OLIG2-*	*IRF7*
		IRF9				*IRF9*	*OLIG2-*
							IRF9

(6B) Transcription factor genes in non-diabetic controls*.

NRES	NLPS	NLPS + RES	NLEC10	NLEC10 + LPS	NLEC50	NLEC50 + LPS

EGR1-	*HDAC9*	*NFKBIA*	*HDAC9*	*HDAC9*	*HDAC9*	*HDAC9*
EGR2-	*PARP12*	*GADD45A*	*NFKBIA*	*PARP12*	*NOTCH3-*	*PARP12*
	NOTCH3 -	*EGR1*	*GADD45A*	***NOTCH3-***	*NFKBIA*	***NOTCH3-***
	NFKBIA	***EGR2-***	*EHF*	*NFKBIA*	*NCOA7*	*NFKBIA*
	KLF5	***IRF5-***	*ETS2*	*NCOA7*	*GADD45A*	*KLF5*
	NCOA7	*EHF*	*E2F7*	*GADD45A*	*EGR1*	*NCOA7*
	GADD45A	*ETS2*	*MAFF*	*TRIM25*	*TRIM5*	*GADD45A*
	TRIM25	*E2F7*		*TRIM5*	*EHF*	*EGR1*
	TRIM5	***ZNF668-***		*EHF*	*PML*	*TRIM25*
	EHF	*HIC1*		*PML*	*ETS2*	*TRIM5*
	PML	**OLIG2-**		*ETS2*	*E2F7*	**TBX3-**
	ETS2			*E2F7*	*MAFF*	*EHF*
	E2F7			*MAFF*	*IRF7*	*PML*
	MAFF			*IRF7*	**OLIG2-**	*ETS2*
	IRF7			***OLIG2-***		*E2F7*
	**OLIG2 -**					*MAFF*
						IRF7
						OLIG2-

*The transcription factor genes that were modulated in the samples. TBX3 (T-box transcription factor 3), NRG1-(Neuroregulin 1), EGR2 (early growth response protein 2), HIC1 (hypermethylated in cancer 1 protein), NFKB1A IκBα (nuclear factor of kappa light polypeptide gene enhancer in B-cells inhibitor, alpha) , GADD45A (growth arrest and DNA-damage-inducible protein 45 alpha), IRF5 (interferon regulatory factor 5), ZNF668 (Zinc finger protein 668), MAFF (v-maf avian musculoaponeurotic fibrosarcoma oncogene homolog F), HDAC9 (histone deacetylase 9), NOTCH3 (Notch gene homolog 3, Drosophila), VDR (vitamin D receptor), TRIM5 (tripartite motif-containing 30, EHF (ets homologous factor), PM (promyelocytic leukemia), ETS2 (E26 avian leukemia oncogene 2, 3' domain), E2F7 (E2F transcription factor 7), IRF7 (interferon regulatory factor 7), OLIG2 (oligodendrocyte transcription factor 2), and IRF9 (interferon regulatory factor 9). **The bold highlighted ones were downregulated,** while all the others were upregulated.
